# Impact of Anaemia on Management and Outcomes in Patients With Atrial Fibrillation: Insights From European and Asian Cohorts

**DOI:** 10.1111/eci.70205

**Published:** 2026-04-18

**Authors:** Andrea Galeazzo Rigutini, Tommaso Bucci, Amir Askarinejad, Enrico Tartaglia, Michele Rossi, Cecilia Becattini, Giuseppe Boriani, Hung‐Fat Tse, Tze‐Fan Chao, Gregory Y. H. Lip

**Affiliations:** ^1^ Liverpool Centre for Cardiovascular Science at University of Liverpool Liverpool John Moores University and Liverpool Heart & Chest Hospital Liverpool UK; ^2^ Internal, Vascular and Emergency Medicine – Stroke Unit University of Perugia Perugia Italy; ^3^ Cardiology Division, Department of Biomedical, Metabolic and Neural Sciences Italy University of Modena and Reggio Emilia, Policlinico di Modena Modena Italy; ^4^ Department of Life, Health & Environmental Sciences University of L'Aquila L'Aquila Italy; ^5^ Internal Medicine and Nephrology Division ASL1 Avezzano‐Sulmona‐L'Aquila, San Salvatore Hospital L'Aquila Italy; ^6^ The University of Hong Kong, Hong Kong Special Administrative Region of China Pok Fu Lam Hong Kong; ^7^ Division of Cardiology, Department of Medicine Taipei Veterans General Hospital Taipei Taiwan; ^8^ Institute of Clinical Medicine and Cardiovascular Research Centre National Yang Ming Chiao Tung University Taipei Taiwan; ^9^ Department of Clinical Medicine Aalborg University Aalborg Denmark; ^10^ Department of Cardiology, Lipidology and Internal Medicine Medical University of Bialystok Bialystok Poland

## Abstract

**Background:**

Anaemia is a common comorbidity in patients with atrial fibrillation (AF) receiving oral anticoagulants (OACs). While its relationship with bleeding is well established, the prothrombotic role and ethnic variations remain unclear.

**Methods:**

We analysed two large prospective AF registries from Europe (EORP‐AF) and East Asia (APHRS‐AF). Patients were classified by anaemia status at enrolment. Logistic regression assessed clinical correlates and treatment patterns, while multivariable Cox models and propensity score matching (PSM) evaluated outcomes. Restricted cubic spline analyses explored the haemoglobin–risk relationship across ethnic groups. The primary outcome was a composite of all‐cause death and major adverse cardiovascular events (MACE); secondary outcomes included individual components and major bleeding (MB).

**Results:**

Among 10,857 patients with AF (mean age 69 ± 11 years, 40.0% women), 3372 (31.0%) had anaemia, which clustered with multimorbidity and frailty. Anaemic patients were less likely to receive OACs (OR 0.67, 95% CI 0.58–0.78) and rhythm‐control (OR 0.91, 95% CI 0.81–1.02). Anaemia was independently associated with higher risk of the composite outcome (HR 1.54, 95% CI 1.34–1.78), all‐cause death (HR 1.81, 95% CI 1.51–2.15), MACE (HR 1.39, 95% CI 1.16–1.66), CV death (HR 1.90, 95% CI 1.43–2.54) and MB (HR 1.79, 95% CI 1.31–2.46) consistent also after PSM. Risk increased progressively with anaemia severity, particularly below 10 g/dL. Associations were consistent across Asian and European cohorts.

**Conclusion:**

Anaemia identifies a vulnerable AF phenotype associated with excess mortality, adverse cardiovascular outcomes and increased bleeding risk, which remains frequently undertreated. Risk rises with anaemia severity, and although biological effects appear consistent across ethnicities, treatment disparities persist. Anaemia should refine, not restrict, therapy within integrated AF care.

## Graphical abstract

Anaemia in atrial fibrillation identifies an undertreated, high‐risk phenotype associated with increased mortality, cardiovascular events, and major bleeding across European and Asian cohorts.

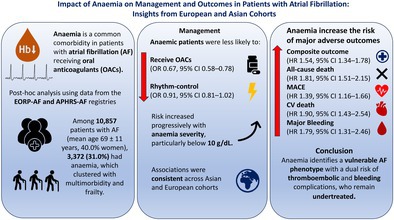

## 1 Introduction

Anaemia is often observed in patients with atrial fibrillation (AF), particularly among elderly individuals and those with multimorbidity and frailty [1, 2]. It is clinically relevant because it is both a marker of bleeding risk and a potential consequence of bleeding, perpetuating a vicious cycle that complicates risk stratification and management [2].

Beyond its haemorrhagic implications, anaemia may also act as a prothrombotic factor. Evidence from other cardiovascular conditions, including coronary artery disease (CAD) [3] and heart failure (HF) [4, 5], suggests associations with ischaemic complications, potentially mediated by impaired oxygen delivery with hypoxia‐driven sympathetic activation, iron deficiency–related platelet hyperreactivity, and oxidative stress and inflammation‐induced endothelial dysfunction, which may promote endothelial activation and atrial remodelling. Whether these mechanisms are clinically relevant in anticoagulated AF remains uncertain [6, 7]. Thus, it is unclear whether anaemia independently contributes to cardiovascular risk in AF.

The complexity is further heightened by ethnic differences in AF presentation, prognosis and management between Asian and European patients [8, 9, 10, 11].

To address this knowledge gap, we analysed pooled data from two large international AF registries based on the same protocol, the EURObservational Research Programme in Atrial Fibrillation General Long‐Term Registry (EORP‐AF; European cohort) [12] and Asia–Pacific Heart Rhythm Society Atrial Fibrillation Registry (APHRS‐AF; Asian cohort) [13], with the following aims: (i) to determine the prevalence and clinical characteristics of anaemia across European and Asian cohorts; (ii) examine differences in management strategies among anaemic patients, including the use of anticoagulation and rhythm‐control strategies; and (iii) evaluate the prognostic impact of anaemia on major adverse outcomes, and assess whether this association varied according to anaemia severity or enrolment setting.

## 2 Methods

### 2.1 Study Design and Population

This is a post hoc analysis of two large prospective observational registries: the EORP‐AF and the APHRS‐AF, which used a common protocol and study procedures, including standardized electronic case report form. Detailed protocols and baseline findings for both registries have been previously reported [12, 13].

Briefly, EORP‐AF enrolled consecutive adult patients (≥ 18 years) with documented AF seen in inpatient or outpatient cardiology settings across 250 centres in 27 European countries between October 2013 and September 2016. The APHRS‐AF registry recruited patients from 52 centres in Hong Kong, Japan, Singapore, South Korea and Taiwan between 2015 and 2017. All patients underwent clinical evaluation by cardiologists and provided written informed consent prior to participation. The EORP‐AF registry was conducted in accordance with the European Union Note for Guidance on Good Clinical Practice (CPMP/ECH/135/95) and the Declaration of Helsinki. The APHRS‐AF study protocol was approved by the local ethics committees of each participating site and was registered on ClinicalTrials.gov (NCT04807049).

### 2.2 Data Collection

Data were collected at baseline using a standardized electronic case report form common to both registries. Collected variables included demographics, medical history, comorbidities, AF characteristics and pharmacological treatments. Follow‐up was conducted prospectively for up to 2 years in EORP‐AF and for 1 year in APHRS‐AF, during which the occurrence of major adverse events—including thromboembolic events, cardiovascular events, bleeding and mortality—was systematically recorded.

### 2.3 Cohort and Definitions

Anaemia was defined according to World Health Organization criteria as a haemoglobin concentration < 13 g/dL in men and < 12 g/dL in women [14]. Rhythm control strategy was defined as the administration of Class Ia, Class Ic, or Class III antiarrhythmic drugs, or the performance of electrical or pharmacological cardioversion, or catheter ablation. Thromboembolic risk was assessed using the CHA_2_DS_2_‐VASc score [15], whereas the bleeding risk was assessed with the HAS‐BLED score [16]. Oral anticoagulation (OACs) use was defined based on prescription at baseline of either vitamin K antagonists or direct oral anticoagulants. According to guidelines contemporaneous to patient enrolment, OACs therapy was indicated for men with CHA_2_DS_2_‐VASc ≥ 1 and women with CHA_2_DS_2_‐VASc ≥ 2 [17, 18].

Patients were stratified according to the presence or absence of anaemia, and for this analysis we included only those with complete data on anaemia status and follow‐up information. In a prespecified sensitivity analysis, patients were grouped by sex‐specific haemoglobin strata—10.0–12.9 g/dL in men and 10.0–11.9 g/dL in women—and severe anaemia defined as < 10.0 g/dL in both sexes; these groups were compared with non‐anaemic patients (≥ 13.0 g/dL in men; ≥ 12.0 g/dL in women).

### 2.4 Outcomes

The primary outcome was a composite of all‐cause death and major adverse cardiovascular events (MACE). MACE was defined as a combination of cardiovascular death (CV death), any acute coronary syndrome (ACS) and thromboembolic events (TEE). All‐cause mortality encompassed death from cardiovascular, non‐cardiovascular, or undetermined causes. Cardiovascular death was defined as death resulting from ACS, HF, arrhythmia, cardiac perforation, tamponade or other unspecified cardiac conditions. Secondary outcomes included the individual components of the primary endpoint (all‐cause mortality and MACE) as well as major bleeding events (MB). MB was defined as the occurrence of intracranial haemorrhage or major extracranial bleeding. Major extracranial bleeding was defined as any bleeding event leading to a haemoglobin decrease > 2 g/dL, requiring blood transfusion, or resulting in hospitalization, involving any major organ system.

### 2.5 Statistical Analysis

Continuous variables were reported as mean ± standard deviation and compared using Student's *t*‐test. Categorical variables were summarized as counts (percentages) and compared using the *χ* ^2^ test.

Baseline comparisons were performed between patients with and without anaemia, and further subgroup summaries as follows.

Univariable and multivariable logistic regression analyses were used to identify factors associated with: (i) presence of anaemia (ii) OACs prescription, and (iii) use of rhythm‐control strategies. Results were expressed as odds ratios (ORs) with 95% confidence intervals (CIs). Multivariable models were adjusted for the following covariates: age, female sex, body mass index (BMI), hypertension, diabetes mellitus, dyslipidaemia, smoking status, dementia, HF, CAD, peripheral artery disease (PAD), prior major bleeding, chronic kidney disease (CKD), prior thromboembolic events, chronic obstructive pulmonary disease, cancer, paroxysmal AF and cohort of recruitment (APHRS vs. EORP) with anaemia status also included for analyses (ii) and (iii).

Incidence rates of clinical outcomes—including all‐cause death, MACE, CV death, ACS, TEE, MB and composite outcomes—were calculated and expressed as events per 100 person‐years, with exact Poisson 95% confidence intervals and between‐group comparisons using the Poisson test.

Univariable and multivariable Cox proportional hazards regression analyses were used to estimate hazard ratios (HRs) and 95% CIs for the risk of adverse clinical outcomes in anaemia patients compared with non‐anaemia patients. Separate multivariable Cox models were constructed for the primary and secondary outcomes, adjusting for age, BMI, female sex, paroxysmal AF, HF, hypertension, diabetes, prior thromboembolic events, PAD, cancer, dementia, CAD, OACs use and region of enrolment (APHRS vs. EORP). The proportional hazards assumption was assessed using Schoenfeld residuals. Kaplan–Meier survival curves were constructed for all clinical outcomes and compared between anaemia and non‐anaemia patients using the log‐rank test.

We conducted four prespecified sensitivity analyses:

- Anaemia severity. Patients were stratified into moderate and severe, with non‐anaemic patients as the reference group. Both the composite outcome and MB were examined, including head‐to‐head comparisons between moderate and severe anaemia.
- Propensity‐score–matched (PSM) analysis. To account for baseline imbalances, anaemic patients were matched 1:1 to non‐anaemic patients using a propensity score estimated from the following baseline covariates: age, sex, BMI, paroxysmal AF, HF, hypertension, diabetes, prior thromboembolic events, PAD, CAD, cancer, dementia, CKD, OACs use and cohort of enrolment. Nearest‐neighbour matching without replacement and a calliper of 0.20 SD of the logit was applied. Balance was evaluated using standardized mean differences (SMD < 0.10 indicating adequate balance). After matching, univariable Cox proportional hazards models were used to estimate the association of anaemia with all clinical outcomes.
- Continuous haemoglobin modelling. Restricted cubic splines (four knots at the 5th, 35th, 65th and 95th percentiles) were used to examine the dose–response association between haemoglobin and outcomes. Analyses were adjusted for age, sex, CHA_2_DS_2_‐VASc score and history of major bleeding. Associations were assessed for the composite outcome, MACE and MB. Non‐linearity was tested with Wald *χ* ^2^ statistics for spline terms. Interaction terms were introduced to assess whether the haemoglobin–risk relationship differed by ethnicity (APHRS vs. EORP), with global tests incorporating both linear and non‐linear components.
- Regional analysis. Univariable and multivariable logistic regression analyses were used to identify factors associated with region (Europe vs. Asia), with results expressed as ORs and 95% CIs. Incidence rates of clinical outcomes were calculated as events per 100 person‐years with exact Poisson 95% CIs. Univariable and multivariable Cox proportional hazards models were then applied to estimate HRs and 95% CIs for the association between region and clinical outcomes, using the same covariates as in the primary analysis.

Exploratory analyses were conducted only in patients with anaemia to assess differences in the risk of composite outcome and MB between patients treated with OACs and those without OACs, and between vitamin K antagonists (VKAs) and non–vitamin K oral anticoagulants (NOACs) users.

To account for the competing risk between non‐fatal cardiovascular events and all cause death, a Fine–Grey competing risk analysis was performed. Non‐fatal cardiovascular events (ACS or TEE) were modelled as the event of interest, with all‐cause death treated as the competing event. Cumulative incidence functions were estimated, and sub distribution hazard ratios (sHRs) with 95% confidence intervals were reported. Multivariable Fine–Grey models were adjusted for the same covariates included in the corresponding multivariable Cox regression models. Subgroup analyses were performed to evaluate potential effect modification of anaemia across clinically relevant strata, including: age (< 80 or ≥ 80 years), sex (male or female), cohort of recruitment (APHRS vs. EORP), obesity status (BMI < 30 or ≥ 30 kg/m^2^), hypertension, PAD, CAD, diabetes, HF, dementia, prior thromboembolism, cancer, CKD, OACs use and rhythm‐control strategies. Results of the subgroup analyses were reported graphically, with HRs and interaction *p*‐values.

All statistical analyses were performed using R version 4.3.1 (R Foundation for Statistical Computing, Vienna, Austria). A two‐sided *p*‐value < 0.05 was considered statistically significant.

## 3 Results

### 3.1 Baseline Characteristics

Among 10,857 patients with AF included in the final study population (Figure S1), 3372 (31.0%) had anaemia at enrolment. Compared with non‐anaemic patients, those with anaemia were older (73 ± 10 vs. 67 ± 12 years; *p* < 0.001), more often female (58.0% vs. 31.0%), and had significantly lower BMI (26.9 ± 5.5 vs. 27.7 ± 5.1). Anaemia was more common among Asian patients than among Europeans (29.0% vs. 27.0%; *p* = 0.03). Patients with anaemia exhibited a broad spectrum of comorbidities—including cardiovascular, renal, metabolic, oncologic and cognitive conditions—which were significantly more prevalent compared with non‐anaemic patients (Table 1).

**TABLE 1:** Baseline characteristics of patients with anaemia and non‐anaemia patients.

Characteristic	Non‐anaemic *N* = 7485	Anaemic *N* = 3372	*p*	Severe anaemia *N* = 374	Moderate anaemia *N* = 2998	*p*
Age, mean ± SD (years)	67 ± 12	73 ± 10	< 0.001	74 ± 11	73 ± 10	0.026
Female	2346 (31.3%)	1950 (57.8%)	< 0.001	193 (51.6%)	1757 (58.6%)	0.011
European	5440 (72.7%)	2385 (70.7%)	0.038	268 (71.7%)	2117 (70.6%)	0.700
Asian	2045 (27.3%)	987 (29.3%)		106 (28.3%)	881 (29.4%)	
BMI	27.7 ± 5.1	26.9 ± 5.5	< 0.001	26.5 ± 5.6	27.0 ± 5.4	0.100
Systolic pressure	132 ± 20	131 ± 21	< 0.001	130 ± 22	131 ± 20	0.400
Diastolic pressure	79 ± 12	75 ± 13	< 0.001	72 ± 13	75 ± 12	< 0.001
Heart rate	81 ± 22	81 ± 23	0.600	82 ± 22	81 ± 23	0.200
Hypertension	4383 (58.6%)	2205 (65.4%)	< 0.001	246 (65.8%)	1959 (65.3%)	0.800
Diabetes mellitus	1515 (20.2%)	1030 (30.5%)	< 0.001	154 (41.2%)	876 (29.2%)	< 0.001
Lipid disorder	3020 (40.4%)	1356 (40.2%)	> 0.900	152 (40.6%)	1204 (40.2%)	0.800
Smoking	809 (10.8%)	167 (5.0%)	< 0.001	22 (5.9%)	145 (4.8%)	0.500
Dementia	73 (1.0%)	92 (2.7%)	< 0.001	10 (2.7%)	82 (2.7%)	> 0.900
Heart failure	2280 (30.5%)	1537 (45.6%)	< 0.001	193 (51.6%)	1344 (44.8%)	0.008
Valvular disease	3150 (42.1%)	2019 (59.9%)	< 0.001	235 (62.8%)	1784 (59.5%)	0.150
Coronary artery disease	1620 (21.6%)	1023 (30.3%)	< 0.001	133 (35.6%)	890 (29.7%)	0.047
Peripheral vascular disease	401 (5.4%)	281 (8.3%)	< 0.001	49 (13.1%)	232 (7.7%)	< 0.001
Pulmonary arterial hypertension	383 (5.1%)	317 (9.4%)	< 0.001	45 (12.0%)	272 (9.1%)	0.200
Aortic plaque	255 (3.4%)	133 (3.9%)	0.300	10 (2.7%)	123 (4.1%)	0.300
COPD	463 (6.2%)	326 (9.7%)	< 0.001	49 (13.1%)	277 (9.2%)	0.022
CKD	595 (8.0%)	633 (18.8%)	< 0.001	120 (32.1%)	513 (17.1%)	< 0.001
Cancer	91 (1.2%)	135 (4.0%)	< 0.001	16 (4.3%)	119 (4.0%)	0.300
Paroxysmal atrial fibrillation	2313 (30.9%)	1028 (30.5%)	0.700	86 (23.0%)	942 (31.4%)	0.002
Prior ischaemic stroke	459 (6.1%)	262 (7.8%)	0.006	23 (6.1%)	239 (8.0%)	0.400
Prior thromboembolic events	837 (11.2%)	460 (13.6%)	< 0.001	50 (13.4%)	410 (13.7%)	> 0.900
Intracranial haemorrhage	80 (1.1%)	54 (1.6%)	0.200	5 (1.3%)	49 (1.6%)	0.600
Prior major extracranial bleeding	154 (2.1%)	160 (4.7%)	< 0.001	39 (10.4%)	121 (4.0%)	< 0.001
Clinically relevant non‐major bleeding	139 (1.9%)	143 (4.2%)	< 0.001	30 (8.0%)	113 (3.8%)	0.002
Aspirin or other antiplatelet drugs	1478 (19.7%)	918 (27.2%)	< 0.001	131 (35.0%)	787 (26.3%)	0.001
VKAs	2844 (38.0%)	1574 (46.7%)	< 0.001	189 (50.5%)	1385 (46.2%)	< 0.001
NOACs	3676 (49.1%)	1242 (36.8%)	< 0.001	281 (75.1%)	2533 (84.5%)	< 0.001
OACs	6517 (87.1%)	2814 (83.4%)	< 0.001	93 (24.9%)	1149 (38.3%)	< 0.001
CHA_2_DS_2_‐VASc score	2.67 ± 1.71	3.80 ± 1.66	< 0.001	4.06 ± 1.61	3.77 ± 1.67	0.001
HAS‐BLED ≥ 3	1021 (13.6%)	843 (25.0%)	< 0.001	145 (38.8%)	698 (23.3%)	< 0.001
Rhythm control	2651 (35.4%)	941 (27.9%)	< 0.001	72 (19.3%)	869 (29.0%)	< 0.001

Abbreviations: AF, atrial fibrillation; BMI, body mass index; CAD, coronary artery disease; CHA_2_DS_2_‐VASc, stroke risk score; CKD, chronic kidney disease; COPD, chronic obstructive pulmonary disease; DBP, diastolic blood pressure; HAS‐BLED, bleeding risk score; HR, heart rate; NOACs, non–vitamin K antagonist oral anticoagulants; OACs, oral anticoagulants; PAD, peripheral artery disease; SBP, systolic blood pressure; VKAs, vitamin K antagonists.

Anaemic patients had a higher burden of prior thrombotic and haemorrhagic events, including ischaemic stroke (7.8% vs. 6.2%), thromboembolic complications (14.0% vs. 11.0%), major extracranial bleeding (14.0% vs. 6.8%), and clinically relevant non‐major bleeding (12.0% vs. 6.2%) (all *p* < 0.001). Their mean CHA_2_DS_2_‐VASc score was significantly higher (3.8 ± 1.7 vs. 2.7 ± 1.7, *p* < 0.001), and the proportion with high bleeding risk (HAS‐BLED ≥ 3) was markedly increased (25.0% vs. 14.0%, *p* < 0.001). Regarding treatment patterns at baseline, patients with anaemia had lower use of OACs compared with those without anaemia (83.0% vs. 87.0%, *p* < 0.001). NOACs were less frequently used in the anaemia group (37% vs. 49%), whereas VKAs had higher use (47.0% vs. 38.0%, both *p* < 0.001). Lastly, rhythm‐control strategies had lower adoption in patients with anaemia compared with non‐anaemic patients (33.0% vs. 42.0%, *p* < 0.001).

### 3.2 Associated Factors of Anaemia and Treatment Patterns Across Ethnic Cohorts

In multivariable analysis, several independent factors associated with anaemia were identified, including older age (OR 1.03, 95% CI 1.03–1.04), female sex (OR 2.92, 95% CI 2.61–3.27), lower BMI (OR 0.97, 95% CI 0.96–0.98), diabetes (OR 1.45, 95% CI 1.28–1.65), HF (OR 1.67, 95% CI 1.49–1.88), CAD (OR 1.43, 95% CI 1.26–1.62), COPD (OR 1.27, 95% CI 1.04–1.56), CKD (OR 1.89, 95% CI 1.60–2.23), cancer (OR 3.20, 95% CI 2.24–4.61) and prior MB (OR 3.52, 95% CI 1.89–6.67) (Table S1).

Anaemia was independently associated with reduced use of OACs (OR 0.67, 0.58–0.78) and with reduced use of rhythm‐control strategies (OR 0.91, 95% CI 0.81–1.02), after adjustment for possible confounders (Tables S2 and S3). Asian patients were confirmed to be more likely to be affected by anaemia compared with Europeans (OR 1.27, 95% CI 1.11–1.44) (Table S1).

### 3.3 Survival Analysis

Among the 3372 patients with anaemia, and after a median follow‐up of 718 days (IQR 368–740), the composite outcome occurred in 541 (16.0%), while all‐cause death in 404 (12.0%), MACE in 314 (9.3%), CV death in 159 (4.7%), ACS in 96 (2.8%), TEE in 72 (2.1%) and MB in 108 (3.2%) (Table 2).

**TABLE 2:** Incidence rates and cox regression analyses for risk of primary and secondary outcomes according to anaemia.

	Number of events	Incidence rate per 100 patient‐years (95% CI)	*p*	Univariable analysis HR (95% CI)	Multivariable analysis HR (95% CI)
Composite outcome					
Anaemia	541	10.63 (9.75–11.56)	< 0.0001	2.29 (2.03–2.57)	1.54 (1.34–1.78)
Non‐anaemia	565	4.64 (4.27–5.04)		Reference	Reference
All‐cause death					
Anaemia	404	7.67 (6.94–8.45)	< 0.0001	2.93 (2.53–3.39)	1.81 (1.51–2.15)
Non‐anaemia	329	2.62 (2.35–2.92)		Reference	Reference
MACE					
Anaemia	314	6.11 (5.45–6.82)	< 0.0001	1.99 (1.71–2.31)	1.39 (1.16–1.66)
Non‐anaemia	375	3.05 (2.75–3.38)		Reference	Reference
Cardiovascular death					
Anaemia	159	3.02 (2.57–3.52)	< 0.0001	3.09 (2.44–3.91)	1.90 (1.43–2.54)
Non‐anaemia	122	0.97 (0.81–1.16)		Reference	Reference
Acute coronary syndrome					
Anaemia	96	1.85 (1.50–2.26)	0.0005	1.58 (1.22–2.05)	1.19 (0.88–1.62)
Non‐anaemia	144	1.16 (0.98–1.37)		Reference	Reference
Thromboembolic events					
Anaemia	72	1.39 (1.09–1.75)	0.092	1.28 (0.96–1.70)	0.92 (0.65–1.30)
Non‐anaemia	133	1.08 (0.91–1.28)		Reference	Reference
Major bleeding					
Anaemia	108	2.09 (1.72–2.53)	< 0.0001	2.32 (1.78–3.02)	1.79 (1.31–2.46)
Non‐anaemia	110	0.88 (0.73–1.07)		Reference	Reference

*Note:* Adjusted for age, female sex, body mass index, paroxysmal AF, heart failure, hypertension, diabetes, prior thromboembolic events, peripheral artery disease, cancer, dementia, coronary artery disease, oral anticoagulant use and region of enrolment (APHRS vs. EORP).

Abbreviations: CI, confidence interval; HR, hazard ratio; MACE, major adverse cardiovascular events.

**FIGURE 1:**
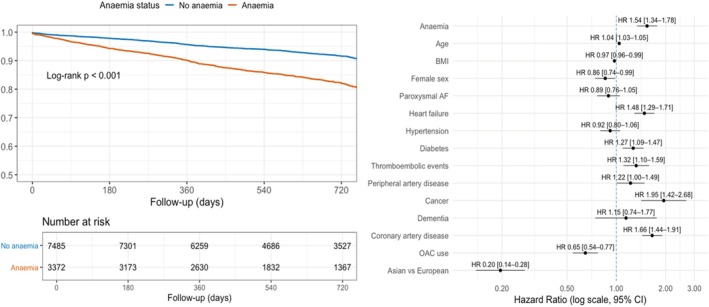
Kaplan–Meier curves and multivariable cox analysis for the composite outcome according to anaemia. The left panel shows Kaplan–Meier estimates of event‐free survival for the composite outcome in patients with (red line) and without anaemia (blue line). Group differences were assessed using the log‐rank test. The right panel displays adjusted hazard ratios (HRs) and 95% confidence intervals (CIs) for the composite outcome derived from the multivariable Cox regression model, including anaemia and all listed covariates. AF, atrial fibrillation; BMI, body mass index; CI, confidence interval; HR, hazard ratio; OAC, oral anticoagulant.

Anaemic patients showed higher incidence rates of both primary and secondary outcomes compared to those without anaemia (Table 2). On univariable Cox regression analysis (Table 2, Figure 1 and Figure S2), patients with anaemia had a higher risk of the composite outcome, all‐cause death, MACE, CV death, ACS and MB compared to those without anaemia.

On multivariable analysis, anaemia remained independently associated with a higher risk of the composite outcome (HR 1.54, 95% CI 1.34–1.78), all‐cause death (HR 1.81, 95% CI 1.51–2.15), MACE (HR 1.39, 95% CI 1.16–1.66), CV death (HR 1.90, 95% CI 1.43–2.54) and MB (HR 1.79, 95% CI 1.31–2.46).

In contrast, the associations with ACS (HR 1.19, 95% CI 0.88–1.62) and TEE (HR 0.92, 95% CI 0.65–1.30) were attenuated and non‐statistically significant (Table 2 and Table S4).

### 3.4 Sensitivity Analyses

#### 3.4.1 Anaemia Severity

**FIGURE 2:**
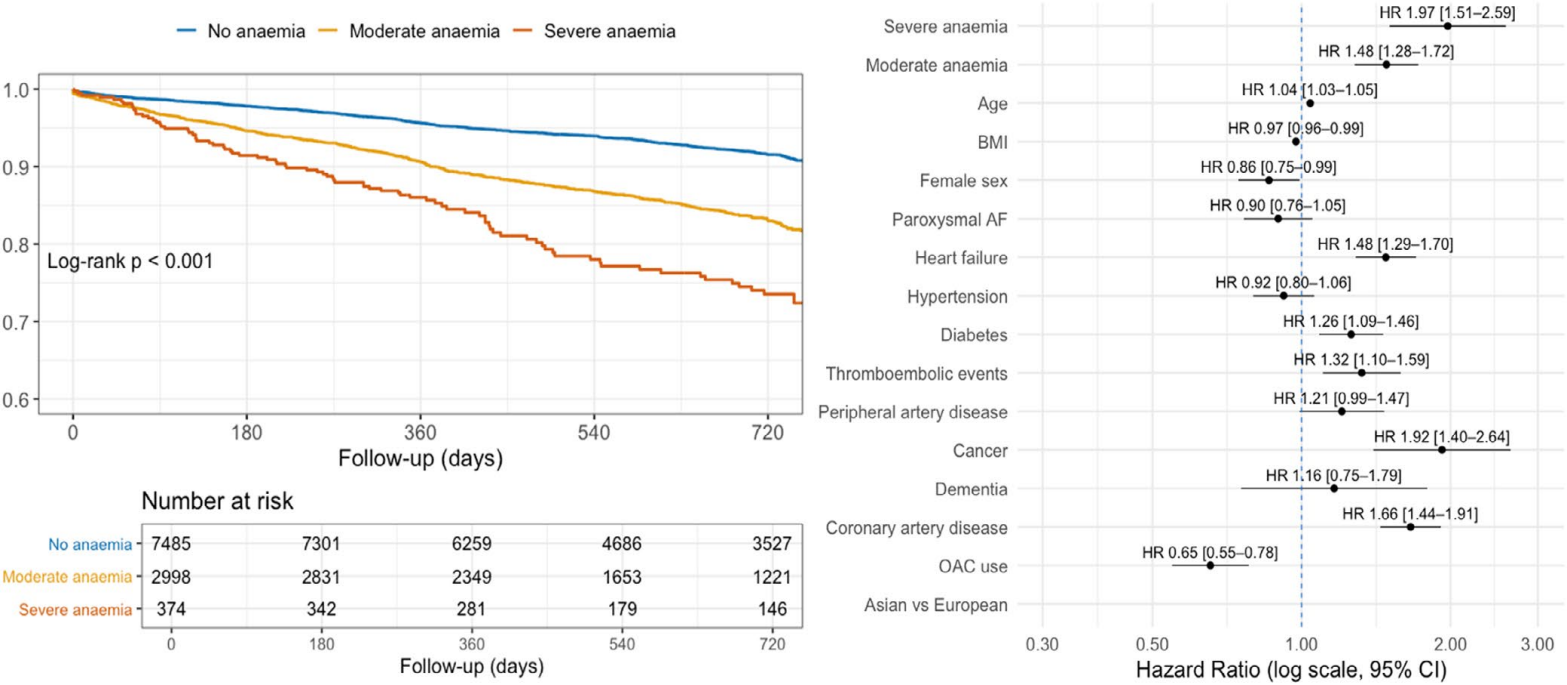
Sensitivity Analysis: Kaplan–Meier curves and multivariable cox model for the composite outcome according to anaemia severity. The left panel shows Kaplan–Meier estimates of event‐free survival for the composite outcome according to anaemia severity: No anaemia (blue line), moderate anaemia (orange line), and severe anaemia (red line). Group differences were assessed using the log‐rank test. The right panel presents adjusted hazard ratios (HRs) and 95% confidence intervals (CIs) for the composite outcome derived from the multivariable Cox regression model, including anaemia severity and all listed covariates. AF, atrial fibrillation; BMI, body mass index; CI, confidence interval; HR, hazard ratio; OAC, oral anticoagulant.

Among 3372 patients with anaemia, 2998 (89.0%) had moderate and 374 (11.0%) had severe anaemia. Patients with severe anaemia were older and had a higher prevalence of diabetes, HF, vascular disease, COPD and CKD, as well as lower use of OACs and rhythm‐control strategies (all *p* < 0.0001), compared with those with moderate anaemia (Table 1). Incidence rates (per 100 patient‐years) for the composite outcome and MB are reported in the Table S5. On multivariable Cox regression, compared with patients without anaemia, both moderate and severe anaemia were associated with a significantly higher risk of the composite outcome and MB (Figure 2 and Figure S3). Direct comparisons showed that patients with severe anaemia had a significantly higher risk of the composite outcome and MB than those with moderate anaemia (Table S5).

#### 3.4.2 Risk of Outcomes After Propensity Score Matching

**FIGURE 3:**
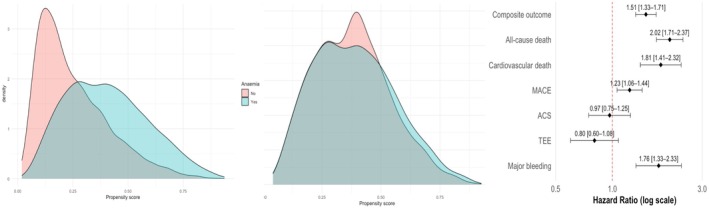
Propensity score matching analysis for anaemia: Covariate balance, propensity score distribution and outcomes. Legend: Propensity score distributions before (left) and after (middle) matching according to anaemia status. Adjusted hazard ratios (HRs) with 95% confidence intervals (CIs) for each outcome are shown on the right. ACS, acute coronary syndrome; CI, confidence interval; HR, hazard ratio; MACE, major adverse cardiovascular events; PSM, propensity score matching; TEE, thromboembolic events.

A total of 2741 patients with anaemia (mean age 72.6 ± 10.4 years, 43.6% female) and 6463 without anaemia (mean age 66.4 ± 11.6 years, 69.4% female) who had complete data for all variables were included in the propensity score analysis (Table S6). Before matching, patients with anaemia were older, more often male, and had a higher burden of comorbidities, including HF, CKD, cancer, and with lower rates of OACs use (Table S6). After 1:1 matching, 2603 well‐balanced pairs were obtained (Figure 3 and Figure S4). On univariable Cox regression analysis after PSM, anaemia remained independently associated with higher risk of the composite outcome (HR 1.51, 95% CI 1.33–1.71), all‐cause death (HR 2.02, 95% CI 1.71–2.37), CV death (HR 1.81, 95% CI 1.41–2.32), MACE (HR 1.23, 95% CI 1.06–1.44) and MB (HR 1.76, 95% CI 1.33–2.33). No significant association was observed for ACS (HR 0.97, 95% CI 0.75–1.25) or TEE (HR 0.80, 95% CI 0.60–1.08) (Figure 3).

#### 3.4.3 Ethnicity and Haemoglobin‐Risk Relationships (Restricted Cubic Splines)

**FIGURE 4:**
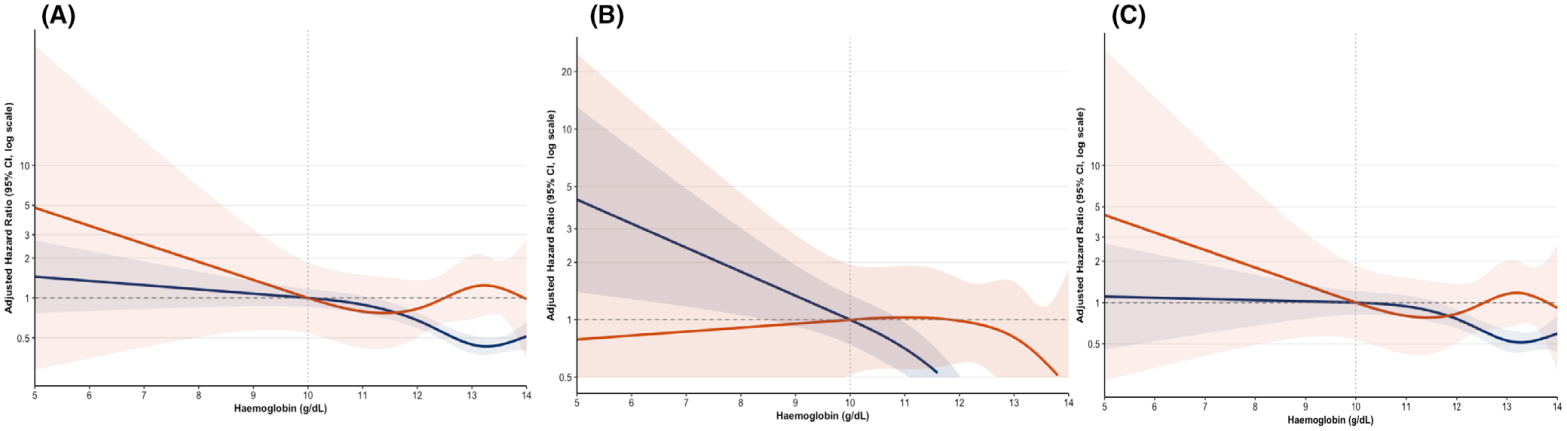
Association of haemoglobin levels with composite outcome, major bleeding and MACE: Adjusted restricted cubic spline analysis by cohort. Restricted cubic spline models show adjusted hazard ratios (HRs) with 95% confidence intervals (CIs) for the associations between haemoglobin and (A) the composite outcome, (B) major bleeding, and (C) MACE, stratified by cohort (APHRS vs. EORP; APHRS shown in red and EORP in blue). At lower haemoglobin levels, risk gradients were steeper in the APHRS cohort, while bleeding risk increased more prominently among EORP patients.

In multivariable‐adjusted models, restricted cubic spline analyses showed that the haemoglobin–risk curves for the composite outcome were non‐linear and similar between the Asian and European cohorts (*p* = 0.14) (Figure 4 and Table S7). Consistently, no statistically significant interactions were found between ethnicity and haemoglobin levels with the risk of MB (*p* = 0.09) and MACE (*p* = 0.31) (Figure 4 and Table S7). Notably, the spline curves showed a clear inflection around a haemoglobin level of approximately 10 g/dL, below which the risk of adverse outcomes increased more markedly.

#### 3.4.4 Differences Between European and Asian Patients With Anaemia

European and Asian patients were of similar age and sex distribution, but showed marked differences in cardiovascular risk profiles (Table S8). VKAs were predominantly used in Europe (56.0% vs. 25.0%), while NOACs use was markedly higher in Asia (60.0% vs. 27.0%) (both *p* < 0.001). Rhythm‐control strategies were slightly more frequent in Europe (34.0% vs. 30.0%, *p* = 0.019). On multivariable analysis, Asian region was independently associated with a lower use of rhythm‐control strategies (OR 0.66, 95% CI 0.53–0.83), whereas regional differences in OACs use were no longer significant after adjustment (Table S9). Event rates were consistently higher in Europe for major cardiovascular outcomes, whereas TEE and MB were similar between regions (Table S10). In multivariable Cox analyses, Asian region was independently associated with lower risk of the composite outcome (HR 0.13, 95% CI 0.07–0.22), MACE (HR 0.24, 95% CI 0.13–0.42) and ACS (HR 0.23, 95% CI 0.08–0.66), with no significant differences for TEE or MB (Table S11).

### 3.5 Exploratory Analyses

#### 3.5.1 Oral Anticoagulant Use in Anaemic Patients

Among anaemic patients, 2814 (75.0%) were treated with OACs and 557 (25.0%) were not treated (Table S12). After adjustment for potential confounders, patients treated with OACs had a lower risk of the composite outcome compared with those not treated (HR 0.61, 95% CI 0.48–0.78) (Figure S5 and Table S13). No significant difference in MB was observed between the two groups (HR 0.80, 95% CI 0.47–1.39) (Figure S6).

#### 3.5.2 Oral Anticoagulant Type (NOACs Versus VKAs) in Anaemic Patients

Among anaemic patients, 1571 (56.0%) received VKAs and 1238 (44.0%) received NOACs. VKAs patients were younger and often European, with a higher prevalence of cardiovascular and renal comorbidities, and higher bleeding risk (Table S12). Among anaemic patients, VKAs use was associated with a higher incidence of the composite outcome and MB compared with NOACs use (Figures S7 and S8 and Table S13). No statistically significant difference was observed after adjustment for possible confounders (Table S13). In anaemic patients, haemoglobin levels were independently associated with the composite outcome (global *p* = 0.019). No significant interaction was observed between haemoglobin and anticoagulant type (*p* for interaction = 0.67) (Table S14). Across the haemoglobin range, VKAs use was generally associated with a higher risk of the composite outcome compared with NOACs, particularly at lower haemoglobin levels. At a haemoglobin value of 10 g/dL, VKAs use was associated with a significantly higher risk compared with NOACs (HR 1.49, 95% CI 1.05–2.12), with attenuation of this difference at higher haemoglobin levels (Figure S9 and Table S15).

### 3.6 Competing Risk Analysis

When accounting for the competing risk of all‐cause death, anaemia was not associated with non‐fatal cardiovascular events. In the Fine‐Grey competing risk model, anaemia was not associated with the cumulative incidence of non‐fatal cardiovascular events (sHR 1.01, 95% CI 0.79–1.29; Figure S10 and Table S16).

### 3.7 Subgroup Analysis

In prespecified subgroup analyses, the association between anaemia and the composite outcome was consistent across most clinical subgroups. However, significant interactions were found in specific strata. The association between anaemia and composite outcomes was stronger among patients with paroxysmal AF compared with those without (*p* for interaction = 0.004), in patients without HF (*p* = 0.002), in those without CAD (*p* = 0.016), among patients not receiving OACs (*p* = 0.014) compared with their counterparts (Figure S11).

## 4 Discussion

In this study, our principal findings are as follows: (i) anaemia was present in approximately 30% of AF patients and was linked to a comorbidity pattern consistent with multimorbidity and frailty; (ii) anaemic patients were less likely to receive OACs and rhythm‐control strategies, especially within the Asian cohort; (iii) anaemia was independently associated with an increased risk of mortality, MACE and MB, an association consistently confirmed after PSM; moreover, the risk of both composite outcome and MB increased progressively with anaemia severity; (iv) restricted cubic spline analyses showed the same impact of haemoglobin lowering in the risk of adverse events between Asians and Europeans; (v) exploratory analyses among anaemic patients suggested improved outcomes with OACs use; and (vi) subgroup analysis revealed greater vulnerability to anaemia among patients without HF or CAD, and those not prescribed OACs.

The first key finding of our analysis is that anaemia was present in approximately 30% of patients with AF, a prevalence consistent with that reported in large contemporary registries [3] and observational studies [1].

Anaemia emerged as a common comorbidity in real‐world AF populations and, in our cohort, was strongly associated with multimorbidity and frailty, reflecting its role as a marker of systemic vulnerability beyond a mere laboratory abnormality [1, 19]. Although anaemia was more prevalent among women, sex did not independently modify the association between anaemia and adverse outcomes after adjustment for clinical confounders, suggesting that the excess risk observed in anaemic patients is largely driven by multimorbidity and overall systemic vulnerability rather than sex‐specific biological differences.

In the context of AF, anaemia appears to serve as a proxy for broader systemic dysfunction, marking a phenotype that is not only biologically vulnerable but also more likely to accumulate both thrombotic and bleeding risk factors [1, 20]. Notably, anaemic patients in our cohort exhibited higher CHA_2_DS_2_‐VASc and HAS‐BLED scores, reinforcing the notion that anaemia coexists with a clustering of adverse prognostic features.

Despite their elevated risk profile, anaemic patients in our cohort were significantly less likely to receive OACs compared with non‐anaemic individuals. However, this pattern may reflect confounding by indication and clinician‐driven risk stratification in the context of higher HAS‐BLED scores and prior bleeding, rather than unequivocal undertreatment, with anaemia acting as a marker of overall clinical vulnerability [1]. Similar patterns have been observed in both Western and Asian cohorts, where anaemic patients were less likely to receive OACs [21, 22]. Importantly, prior evidence indicates that anaemia should not be regarded as a contraindication to anticoagulation, as NOACs maintain a favourable net clinical benefit even in patients with anaemia [20, 23]. In line with this, previous studies have highlighted the potential consequences of restrictive use of OACs driven by bleeding concerns. In the AFIRE trial [24], anticoagulant‐based strategies were associated with improved survival despite high bleeding risk, while real‐world evidence from the COOL‐AF registry [25] showed that withholding OAC therapy in high‐risk AF patients was associated with increased mortality and thromboembolic events. In our cohort, rhythm‐control strategies were also less frequently used in anaemic patients. This pattern may reflect a broader perception of clinical vulnerability. Such treatment patterns have been reported in other AF cohorts and likely reflect the complex balance between thromboembolic prevention, bleeding risk and complexity in patients with multiple comorbidities. While our observational design precludes causal inference, these findings highlight the presence of variability in therapeutic decision‐making and suggest that anaemia may influence clinicians' management strategies in patients with AF.

Notably, differences in treatment patterns were particularly evident among Asian patients, who were less likely to receive rhythm‐control interventions and OAC therapy, consistent with previous reports from these registries [9, 26]. Such differences may reflect not only clinical characteristics but also system‐level and contextual factors, including differences in healthcare infrastructure, reimbursement policies, drug availability and physician perceptions of bleeding risk [27, 28, 29]. Taken together, these findings highlight the need for a consistent application of evidence‐based treatment strategies.

In our analyses, anaemia was independently associated with a higher risk of both MACE and MB, with these associations consistently confirmed after PSM. While previous AF registries have consistently shown an excess bleeding risk among anaemic patients, evidence regarding thromboembolic complications has been less consistent [1, 30, 31, 32, 33]. In the present cohort, the prognostic signal associated with anaemia was largely driven by fatal events and bleeding, underscoring its relevance beyond traditional clinical confounders. When the composite endpoint was disaggregated, anaemia remained associated with MACE, whereas associations with individual non‐fatal cardiovascular events, including ACS and TEE, were weaker and less consistent. Due to lower statistical power associated with analysing each component of MACE separately, these results should not be interpreted as evidence against a cardiovascular risk signal related to anaemia. Rather, in this frail and multimorbid population, the substantial burden of mortality represents a dominant competing event that may attenuate the occurrence of non‐fatal cardiovascular outcomes. Competing risk analyses are consistent with this interpretation, showing that differences in the cumulative incidence of non‐fatal events according to anaemia status do not clearly emerge once the competing risk of death is taken into account.

Importantly, anaemia is not a uniform entity: haemoglobin thresholds identify its presence but do not capture the underlying aetiology, which may itself influence cardiovascular vulnerability. Iron‐deficiency anaemia may amplify thrombotic propensity through impaired oxygen delivery, sympathetic activation and heightened platelet reactivity, whereas anaemia of chronic disease often reflects a pro‐inflammatory milieu characterized by immune activation, oxidative stress and altered erythropoiesis [7]. In parallel, systemic inflammation and iron deficiency may contribute to atrial remodelling and adverse myocardial structural changes, predisposing to both AF progression and HF [34, 35]. Consistent with this biological heterogeneity, different anaemia subtypes may be associated with distinct prognostic patterns, suggesting that consideration of the underlying cause could help refine risk stratification and may assist in identifying whether correction of potentially reversible forms might mitigate adverse outcomes in patients with AF.

Our subgroup analysis further demonstrated that the prognostic effect of anaemia was greater in patients without HF or CAD, suggesting that in lower‐risk individuals, anaemia marks a transition towards greater systemic vulnerability. In contrast, in those with advanced comorbidities, its impact may be masked by competing mechanisms driving poor outcomes. These findings highlight the nuanced and context‐dependent nature of anaemia's prognostic role in AF, reflecting a complex interplay between systemic oxygen delivery, cardiovascular reserve and underlying disease burden.

Our analyses showed that at lower haemoglobin levels, Asian patients exhibited a comparable increase in the risk of composite outcomes to that observed in Europeans [32, 36]. Similar patterns were confirmed for both MACE and MB. These findings suggest that, despite well‐recognized ethnic differences, the biological consequences of declining haemoglobin levels may impose a similar degree of physiological stress across different ethnic groups. Consequently, differences in adverse event risk between Asian and European patients in our study may reflect differences in baseline clinical profiles, comorbidity burden, treatment patterns and follow‐up duration, rather than inherent biological differences alone. Although our analyses accounted for follow‐up time using time‐to‐event models, residual differences related to the unequal duration of observation between registries cannot be completely excluded.

Patients with severe anaemia showed a clear gradient of risk, with higher rates of the composite outcome and MB compared with those with moderate anaemia, confirming a clear gradient of risk. The restricted cubic spline analyses identified a critical inflection approximately around 10 g/dL—corresponding to the transition from moderate to severe anaemia—beyond which adverse outcomes rose steeply. These findings suggest that accounting for anaemia severity, rather than its mere presence, may improve prognostic precision and help identify patients requiring closer monitoring or tailored anticoagulation strategies. Future risk stratification therefore benefits from incorporating haemoglobin severity to capture this additional layer of clinical vulnerability.

Consistently with previous studies, our exploratory analysis suggested that OACs use was associated with a significantly lower risk of the composite outcome, without a corresponding increase in MB among anaemic patients [20, 22, 37, 38]. However, given the observational nature of the analysis, these results should not imply causality but simply associations. Indeed, they should be interpreted with caution and may reflect differences in clinical decision‐making and patient risk profiles rather than a direct causal effect of treatment.

For patients in whom bleeding risk is high, alternative strategies warrant consideration. Left atrial appendage closure has emerged as a non‐pharmacological approach that can provide stroke protection comparable to OACs, and its role may be particularly relevant in anaemic or bleeding‐prone patients [39].

In selected cases, optimization of reversible contributors to anaemia and closer haemoglobin surveillance may further support safer implementation of stroke‐prevention strategies. In parallel, pharmacological innovations are beginning to reshape the therapeutic landscape.

Very low‐dose regimens of currently available anticoagulants, as tested in selected high‐risk populations, have shown promising results [40, 41], while novel agents such as factor XI inhibitors may offer effective thromboprophylaxis with reduced bleeding liability [42]. Together, these approaches raise the concept of haemoglobin‐informed or phenotype‐guided anticoagulation, which merits prospective evaluation. Although not yet part of routine practice, these developments illustrate the evolving range of options that may allow safer stroke prevention in vulnerable AF populations.

In this context, anaemia should prompt careful risk–benefit assessment, but not therapeutic nihilism. Rather, it should guide clinicians towards more nuanced strategies—including closer monitoring, individualized treatment intensity, and correction of reversible causes of anaemia—balancing the established benefits of anticoagulation with the tailored use of emerging alternatives within the broader framework of integrated care. Contemporary guidelines increasingly emphasize holistic management, most notably the ABC pathway [43], whose adherence has been associated with significant reductions in mortality, hospitalization and adverse events [44, 45, 46, 47]. More recent conceptual acronym refinements, such as AF‐CARE [48] and SOS [49], further highlight that optimal management of complex AF populations requires not only better drugs or devices, but coordinated, multidisciplinary care [50].

### 4.1 Limitations

This study has some limitations. First, it represents a post hoc analysis of two prospective, observational registries, and is therefore subject to selection bias, residual confounding and misclassification. Given the post hoc observational nature of the analysis, residual confounding and reverse causation cannot be fully excluded. Accordingly, anaemia should be interpreted as a clinically relevant marker of systemic vulnerability and risk, rather than as a definitive causal determinant of adverse outcomes. Second, treatment exposure was only assessed at baseline; changes in anticoagulation or rhythm control strategy during follow‐up were not captured. Furthermore, observed differences in anticoagulation or rhythm‐control strategies between groups may reflect confounding by indication and clinician decision‐making rather than causal effects on outcomes. Third, patients with missing haemoglobin values and those without available or valid follow‐up for the investigated outcomes were excluded, resulting in a complete‐case analysis that may have introduced selection bias. Fourth, another limitation of this study is the unequal follow‐up duration between the two registries (EORP‐AF, 2 years; APHRS‐AF, 1 year) which may have influenced event ascertainment. Fifth, haemoglobin was measured only at baseline, and no longitudinal assessments were available. We were therefore unable to evaluate temporal changes, correction of anaemia, or the impact of therapeutic interventions on subsequent risk factors that may substantially modify prognosis. Moreover, the registries did not capture the underlying aetiology of anaemia, which is inherently heterogeneous and may carry distinct clinical implications. Iron‐deficiency anaemia, inflammation‐driven anaemia, renal‐related erythropoietin deficiency and occult blood loss represent biologically different states that may influence cardiovascular vulnerability in disparate ways. The absence of aetiological data prevented us from performing subtype‐specific analyses, which would be valuable to refine prognostic interpretation. Future studies incorporating serial haemoglobin measurements and detailed anaemia phenotyping are needed to address these knowledge gaps. Finally, analyses of OACs in anaemic patients were exploratory and subject to confounding by indication. Comparisons between NOACs and VKAs were limited by sample size and should be interpreted with caution, reflecting potential differences in prescribing practices across regions. In addition, a substantial proportion of anaemic patients not receiving OAC therapy were treated with antiplatelet agents. Although this may reflect attempts to provide an alternative antithrombotic strategy in frail patients, current evidence indicates that antiplatelet therapy provides substantially less protection against thromboembolic events while conferring a broadly comparable bleeding risk in patients with AF.

## 5 Conclusion

Anaemia identifies a vulnerable subset of AF patients characterized by multimorbidity, increased bleeding risk and adverse outcomes, who remain frequently undertreated. Risk increases progressively with anaemia severity, and although the direction of effect appears consistent across ethnic groups, differences in treatment patterns persist. Anaemia should therefore refine, not restrict, therapy within integrated, patient‐centred models of AF care.

## Author Contributions

A.G.R. and T.B. conceived and designed the study, performed the statistical analyses, interpreted the data and drafted the manuscript. A.A., E.T. and M.R. contributed to data interpretation and critically revised the manuscript. C.B., G.B. and H.‐F.T. contributed to study conception and clinical interpretation of the findings. T.‐F.C. and G.Y.H.L. supervised the study, contributed to study design and interpretation of results, and critically revised the manuscript.

## Funding

The authors have nothing to report.

## Conflicts of Interest

G.B. is the Principal Investigator of the ARISTOTELES project (Applying ARtificial Intelligence to define clinical trajectorieS for personalized predicTiOn and early deTEction of comorbidity and muLti‐morbidity pattErnS) that received funding from the European Union within the Horizon 2020 research and innovation programme (grant no. 101080189). G.Y.H.L. has been a consultant and speaker for BMS/Pfizer, Boehringer Ingelheim, Anthos and Daiichi‐Sankyo. No fees are directly received personally. All the disclosures happened outside the submitted work. He is a National Institute for Health and Care Research (NIHR) Senior Investigator and co‐PI of the AFFIRMO project on multimorbidity in AF (grant agreement No 899871), TARGET project on digital twins for personalized management of atrial fibrillation and stroke (grant agreement No 101136244), and ARISTOTELES project on artificial intelligence for management of chronic long‐term conditions (grant agreement No 101080189), which are all funded by the EU's Horizon Europe Research and Innovation program. The other authors did not report conflicts of interest to disclose outside of the submitted work.

## Supporting information


**Table S1:** Univariable and multivariable logistic regression for factors associated with anaemia.
**Table S2:** Univariable and multivariable logistic regression for factors associated with oral anticoagulants use.
**Table S3:** Univariable and multivariable logistic regression for factors associated with Rhythm control strategies.
**Table S4:** Multivariable Cox regression analysis for factors associated with primary and secondary outcomes.
**Table S5:** Incidence rates and adjusted hazard ratios for the composite outcome and major bleeding according to anaemia severity.
**Table S6:** Baseline characteristics of patients with and without anaemia before and after propensity score matching.
**Table S7:** Interaction between haemoglobin levels and ethnicity for clinical outcomes (multivariable RCS models).
**Table S8:** Baseline characteristics of anaemic patients according to enrolment setting.
**Table S9:** Univariable and multivariable logistic regression for factors associated with enrolment setting.
**Table S10:** Incidence rates for the outcomes to enrolment setting.
**Table S11:** Adjusted hazard ratios for the outcomes according to enrolment setting.
**Table S12:** Baseline characteristics of anaemic patients according to anticoagulation status and type of oral anticoagulant (NOACs vs VKAs).
**Table S13:** Incidence rates and adjusted hazard ratios for the composite outcome and major bleeding according to exploratory analyses.
**Table S14:** Global and interaction tests for haemoglobin and anticoagulant type in anaemic patients.
**Table S15:** VKAs versus NOACs according to haemoglobin levels in anaemic patients.
**Table S16:** Fine–Grey competing risk analysis for non‐fatal cardiovascular events.
**Figure S1:** Flow chart.
**Figure S2:** Kaplan–Meier curves for the secondary outcomes according to anaemia.
**Figure S3:** Sensitivity analysis: Kaplan–Meier curves and multivariable Cox model for the major bleeding according to anaemia severity.
**Figure S4:** Covariate balance before and after propensity score matching (PSM).
**Figure S5:** Exploratory analysis: Kaplan–Meier curves and multivariable Cox model for the composite outcome according to anticoagulation status (OACs vs. no‐OACs).
**Figure S6:** Exploratory analysis: Kaplan–Meier curves and multivariable Cox model for the major bleeding according to anticoagulation status (OACs vs. no‐OACs).
**Figure S7:** Exploratory analysis: Kaplan–Meier curves and multivariable Cox Model for the Composite outcome according to type of anticoagulant (NOACs vs VKAs).
**Figure S8:** Exploratory Analysis: Kaplan–Meier curves and multivariable Cox model for the major bleeding according to type of anticoagulant (NOACs vs VKAs).
**Figure S9:** Association between haemoglobin levels and composite outcome according to anticoagulant type in anaemic patients (NOACs vs VKAs).
**Figure S10:** Competing risk analysis of fatal and non‐fatal cardiovascular events.
**Figure S11:** Effect of anaemia on the composite outcome in prespecified subgroups.

## Data Availability

The data that support the findings of this study are available from the corresponding author upon reasonable request.
