# Supplementary material for: Impact of Anaemia on Management and Outcomes in Patients With Atrial Fibrillation: Insights From European and Asian Cohorts
Source: Eur J Clin Invest. 2026 Apr 18;56:e70205. doi: 10.1111/eci.70205 (PMC13091571; doi:10.1111/eci.70205)
Supplement: Supplementary file 1 — Table S1: Univariable and multivariable logistic regression for factors associated with anaemia. Table S2: Univariable and multivariable logistic regression for factors associated with oral anticoagulants use. Table S3: Univariable and multivariable logistic regression for factors associated with Rhythm control strategies. Table S4: Multivariable Cox regression analysis for factors associated with primary and secondary outcomes. Table S5: Incidence rates and adjusted hazard ratios for the composite outcome and major bleeding according to anaemia severity. Table S6: Baseline characteristics of patients with and without anaemia before and after propensity score matching. Table S7: Interaction between haemoglobin levels and ethnicity for clinical outcomes (multivariable RCS models). Table S8: Baseline characteristics of anaemic patients according to enrolment setting. Table S9: Univariable and multivariable logistic regression for factors associated with enrolment setting. Table S10: Incidence rates for the outcomes to enrolment setting. Table S11: Adjusted hazard ratios for the outcomes according to enrolment setting. Table S12: Baseline characteristics of anaemic patients according to anticoagulation status and type of oral anticoagulant (NOACs vs VKAs). Table S13: Incidence rates and adjusted hazard ratios for the composite outcome and major bleeding according to exploratory analyses. Table S14: Global and interaction tests for haemoglobin and anticoagulant type in anaemic patients. Table S15: VKAs versus NOACs according to haemoglobin levels in anaemic patients. Table S16: Fine–Grey competing risk analysis for non‐fatal cardiovascular events. Figure S1: Flow chart. Figure S2: Kaplan–Meier curves for the secondary outcomes according to anaemia. Figure S3: Sensitivity analysis: Kaplan–Meier curves and multivariable Cox model for the major bleeding according to anaemia severity. Figure S4: Covariate balance before and after propensity score matching (PSM). Figure S5: [file ECI-56-e70205-s001.docx]

**Supplementary Methods.**

Andrea Galeazzo Rigutini, Tommaso Bucci, Amir Askarinejad, Enrico Tartaglia, Michele Rossi,

Cecilia Becattini, Giuseppe Boriani, Hung-Fat Tse, Tze-Fan Chao, Gregory Y. H. Lip

**Supplementary Table 1. Univariable and multivariable logistic regression for factors associated with anaemia.**

| Variable | Univariate OR (95% CI) | p | Multivariate OR (95% CI) | p |
| --- | --- | --- | --- | --- |
| Age | 1.055 (1.051–1.060) | <0.001 | 1.033 (1.027–1.039) | <0.001 |
| Female | 3.00 (2.76–3.27) | <0.001 | 2.92 (2.61–3.27) | <0.001 |
| BMI | 0.97 (0.97–0.98) | <0.001 | 0.97 (0.96–0.98) | <0.001 |
| Hypertension | 1.34 (1.23–1.46) | <0.001 | 1.01 (0.90–1.14) | 0.885 |
| Diabetes | 1.74 (1.59–1.91) | <0.001 | 1.45 (1.28–1.65) | <0.001 |
| Dyslipidaemia | 1.00 (0.92–1.09) | 0.972 | 0.83 (0.74–0.94) | 0.002 |
| Smoking | 0.43 (0.36–0.51) | <0.001 | 0.57 (0.45–0.73) | <0.001 |
| Dementia | 2.85 (2.09–3.89) | <0.001 | 1.12 (0.72–1.72) | 0.620 |
| Heart failure | 1.93 (1.78–2.10) | <0.001 | 1.67 (1.49–1.88) | <0.001 |
| CAD | 1.62 (1.48–1.78) | <0.001 | 1.43 (1.26–1.62) | <0.001 |
| PAD | 1.61 (1.38–1.89) | <0.001 | 1.12 (0.91–1.39) | 0.276 |
| COPD | 1.63 (1.40–1.88) | <0.001 | 1.27 (1.04–1.56) | 0.020 |
| CKD | 2.68 (2.37–3.02) | <0.001 | 1.89 (1.60–2.23) | <0.001 |
| Thromboembolic events | 1.26 (1.11–1.42) | <0.001 | 1.03 (0.87–1.20) | 0.763 |
| Cancer | 3.40 (2.60–4.45) | <0.001 | 3.20 (2.24–4.61) | <0.001 |
| Paroxysmal AF | 0.98 (0.90–1.07) | 0.689 | 1.15 (1.02–1.30) | 0.020 |
| Prior Major bleeding | 4.04 (2.47–6.59) | <0.001 | 3.52 (1.89–6.67) | <0.001 |
| OACs | 0.75 (0.67–0.84) | <0.001 | 0.88 (0.79-0.97) | <0.001 |
| Rhythm control | 0.67 (0.61–0.74) | <0.001 | 0.91 (0.81–1.03) | 0.124 |
| Asian cohort (APHRS) | 1.10 (1.01–1.20) | 0.036 | 1.27 (1.11–1.44) | <0.001 |

*Abbreviations: AF, atrial fibrillation; BMI, body mass index; CAD, coronary artery disease; PAD, peripheral artery disease; CKD, chronic kidney disease; COPD, chronic obstructive pulmonary disease; OR, odds ratio; CI, confidence interval*

**Supplementary Table 2. Univariable and multivariable logistic regression for factors associated with oral anticoagulants use.**

| Variable | Univariate OR (95% CI) | p | Multivariate OR (95% CI) | p |
| --- | --- | --- | --- | --- |
| Age | 1.023 (1.018–1.027) | <0.001 | 1.029 (1.023–1.035) | <0.001 |
| Female | 0.98 (0.88–1.10) | 0.729 | 1.02 (0.89–1.18) | 0.758 |
| BMI | 1.03 (1.02–1.04) | <0.001 | 1.03 (1.01–1.04) | <0.001 |
| Anaemia | 0.75 (0.67–0.84) | <0.001 | 0.67 (0.58–0.78) | <0.001 |
| Hypertension | 1.59 (1.42–1.77) | <0.001 | 1.41 (1.23–1.61) | <0.001 |
| Diabetes | 1.22 (1.07–1.39) | 0.003 | 1.10 (0.93–1.30) | 0.286 |
| Dyslipidaemia | 1.24 (1.11–1.39) | <0.001 | 1.18 (1.03–1.36) | 0.018 |
| Smoking | 0.73 (0.61–0.87) | <0.001 | 0.84 (0.68–1.03) | 0.086 |
| Dementia | 0.38 (0.27–0.53) | <0.001 | 0.23 (0.15–0.36) | <0.001 |
| Heart failure | 1.13 (1.01–1.27) | 0.032 | 1.12 (0.96–1.30) | 0.140 |
| CAD | 0.76 (0.68–0.86) | <0.001 | 0.64 (0.55–0.75) | <0.001 |
| PAD | 0.91 (0.73–1.13) | 0.408 | 0.80 (0.62–1.05) | 0.104 |
| COPD | 1.21 (0.97–1.51) | 0.097 | 1.16 (0.89–1.54) | 0.282 |
| CKD | 0.83 (0.71–0.98) | 0.026 | 0.75 (0.61–0.93) | 0.008 |
| Thromboembolic events | 1.79 (1.47–2.19) | <0.001 | 1.98 (1.56–2.56) | <0.001 |
| Cancer | 0.48 (0.35–0.64) | <0.001 | 0.40 (0.28–0.58) | <0.001 |
| Paroxysmal AF | 0.53 (0.48–0.60) | <0.001 | 0.58 (0.50–0.66) | <0.001 |
| Prior Major bleeding | 0.29 (0.18–0.48) | <0.001 | 0.29 (0.16–0.52) | <0.001 |
| Rhythm control | 1.02 (0.90–1.15) | 0.813 | 1.11 (0.95-1.29) | 0.289 |
| Asian cohort (APHRS) | 0.99 (0.88–1.11) | 0.838 | 1.11 (0.95–1.29) | 0.191 |

*Abbreviations: AF, atrial fibrillation; BMI, body mass index; CAD, coronary artery disease; PAD, peripheral artery disease; CKD, chronic kidney disease; COPD, chronic obstructive pulmonary disease; OACs, oral anticoagulants; OR, odds ratio; CI, confidence interval*

**Supplementary Table 3. Univariable and multivariable logistic regression for factors associated with Rhythm control strategies.**

| Variable | Univariate OR (95% CI) | p | Multivariate OR (95% CI) | p |
| --- | --- | --- | --- | --- |
| Age | 0.97 (0.96–0.97) | <0.001 | 0.98 (0.97–0.98) | <0.001 |
| Female | 0.94 (0.86–1.02) | 0.125 | 1.01 (0.90–1.12) | 0.922 |
| BMI | 1.01 (1.00–1.02) | 0.054 | 1.00 (0.99–1.01) | 0.476 |
| Anaemia | 0.67 (0.61–0.74) | <0.001 | 0.91 (0.81–1.02) | 0.107 |
| Hypertension | 0.73 (0.67–0.79) | <0.001 | 0.93 (0.84–1.04) | 0.199 |
| Diabetes | 0.59 (0.54–0.66) | <0.001 | 0.73 (0.64–0.82) | <0.001 |
| Dyslipidaemia | 0.92 (0.84–1.00) | 0.046 | 1.10 (0.99–1.22) | 0.074 |
| Smoking | 1.26 (1.09–1.47) | 0.002 | 0.98 (0.82–1.17) | 0.816 |
| Dementia | 0.36 (0.24–0.54) | <0.001 | 0.51 (0.29–0.85) | 0.014 |
| Heart failure | 0.49 (0.45–0.53) | <0.001 | 0.62 (0.56–0.70) | <0.001 |
| CAD | 0.69 (0.63–0.77) | <0.001 | 0.86 (0.76–0.97) | 0.015 |
| PAD | 0.59 (0.49–0.71) | <0.001 | 0.82 (0.66–1.02) | 0.071 |
| COPD | 0.64 (0.54–0.76) | <0.001 | 0.83 (0.68–1.02) | 0.079 |
| CKD | 0.58 (0.51–0.67) | <0.001 | 0.89 (0.75–1.06) | 0.182 |
| Thromboembolic events | 0.81 (0.71–0.93) | 0.002 | 0.93 (0.80–1.08) | 0.333 |
| Cancer | 0.53 (0.38–0.74) | <0.001 | 0.58 (0.39–0.86) | 0.008 |
| Paroxysmal AF | 2.46 (2.25–2.69) | <0.001 | 2.34 (2.11–2.60) | <0.001 |
| Prior Major bleeding | 1.05 (0.62–1.79) | 0.857 | 1.28 (0.68–2.36) | 0.428 |
| OACs | 1.02 (0.90–1.15) | 0.813 | 1.11 (0.88-1.29) | 0.326 |
| Asian ethnicity (APHRS) | 0.76 (0.69–0.84) | <0.001 | 0.56 (0.49–0.63) | <0.001 |

*Abbreviations: AF, atrial fibrillation; BMI, body mass index; CAD, coronary artery disease; PAD, peripheral artery disease; CKD, chronic kidney disease; COPD, chronic obstructive pulmonary disease; OACs, oral anticoagulants; OR, odds ratio; CI, confidence interval*

**Supplementary Table 4. Multivariable Cox Regression Analysis for Factors Associated with Primary and Secondary Outcomes.**

| **Variables** | **Composite** | **MACE** | **All-cause of death** | **CV death** | **ACS** | **TTE** | **MB** |
| --- | --- | --- | --- | --- | --- | --- | --- |
|  | **Multivariable**  **HR [95% CI]** | **Multivariable**  **HR [95% CI]** | **Multivariable**  **HR [95% CI]** | **Multivariable**  **HR [95% CI]** | **Multivariable**  **HR [95% CI]** | **Multivariable**  **HR [95% CI]** | **Multivariable**  **HR [95% CI]** |
| **Anaemia** | 1.54 (1.34–1.78) | 1.81 (1.51–2.15) | 1.39 (1.16–1.66) | 1.90 (1.43–2.54) | 1.19 (0.88–1.62) | 0.92 (0.65–1.31) | 1.79 (1.31–2.46) |
| **Age** | 1.04 (1.03–1.05) | 1.06 (1.05–1.08) | 1.03 (1.02–1.03) | 1.05 (1.03–1.07) | 1.01 (1.00–1.03) | 1.01 (1.00–1.03) | 1.02 (1.01–1.04) |
| **BMI** | 0.97 (0.96–0.99) | 0.97 (0.95–0.98) | 0.97 (0.96–0.99) | 0.95 (0.92–0.98) | 0.99 (0.96–1.03) | 0.98 (0.95–1.01) | 0.99 (0.96–1.02) |
| **Female** | 0.86 (0.74–0.99) | 0.78 (0.65–0.93) | 0.91 (0.76–1.09) | 0.76 (0.57–1.02) | 0.89 (0.65–1.21) | 1.19 (0.86–1.65) | 0.84 (0.61–1.14) |
| **Paroxysmal AF** | 0.89 (0.76–1.05) | 0.73 (0.59–0.90) | 0.97 (0.79–1.18) | 0.61 (0.42–0.89) | 1.26 (0.93–1.70) | 1.11 (0.79–1.56) | 1.00 (0.71–1.39) |
| **Heart Failure** | 1.48 (1.29–1.71) | 1.76 (1.47–2.09) | 1.59 (1.33–1.91) | 2.93 (2.13–4.01) | 1.06 (0.79–1.42) | 1.36 (0.98–1.89) | 1.70 (1.25–2.32) |
| **Hypertension** | 0.92 (0.80–1.06) | 0.88 (0.74–1.05) | 0.94 (0.78–1.12) | 0.84 (0.63–1.12) | 0.96 (0.71–1.30) | 1.12 (0.80–1.56) | 0.77 (0.57–1.04) |
| **Diabetes** | 1.27 (1.09–1.47) | 1.36 (1.13–1.63) | 1.30 (1.08–1.57) | 1.53 (1.15–2.05) | 0.96 (0.70–1.34) | 1.52 (1.07–2.16) | 1.04 (0.74–1.46) |
| **Thromboembolic events** | 1.32 (1.10–1.59) | 1.24 (0.99–1.55) | 1.49 (1.19–1.87) | 1.35 (0.94–1.95) | 1.14 (0.76–1.72) | 2.06 (1.41–3.02) | 1.42 (0.97–2.10) |
| **PAD** | 1.22 (1.00–1.49) | 1.42 (1.13–1.78) | 1.03 (0.79–1.35) | 1.38 (0.97–1.98) | 0.66 (0.39–1.13) | 0.92 (0.52–1.62) | 1.00 (0.60–1.67) |
| **Cancer** | 1.95 (1.42–2.68) | 2.53 (1.78–3.60) | 1.36 (0.85–2.20) | 1.65 (0.84–3.26) | 0.42 (0.10–1.70) | 2.09 (0.97–4.50) | 3.50 (2.04–6.00) |
| **Dementia** | 1.15 (0.74–1.77) | 1.35 (0.84–2.15) | 0.91 (0.50–1.68) | 1.03 (0.47–2.24) | 0.29 (0.04–2.07) | 1.04 (0.33–3.34) | 1.52 (0.61–3.78) |
| **CAD** | 1.66 (1.44–1.91) | 1.33 (1.12–1.59) | 2.06 (1.73–2.47) | 1.68 (1.27–2.23) | 5.57 (4.06–7.65) | 0.85 (0.59–1.23) | 0.99 (0.71–1.38) |
| **OACs** | 0.65 (0.54–0.77) | 0.69 (0.55–0.87) | 0.58 (0.47–0.72) | 0.55 (0.39–0.78) | 0.58 (0.41–0.81) | 0.56 (0.37–0.83) | 1.06 (0.68–1.65) |
| **Asian cohorts** | 0.47 (0.38–0.57) | 0.47 (0.37–0.61) | 0.43 (0.32–0.57) | 0.30 (0.18–0.49) | 0.42 (0.25–0.70) | 0.54 (0.32–0.90) | 0.70 (0.46–1.08) |

**Supplementary Table 5.** **Incidence rates and adjusted hazard ratios for the composite outcome and major bleeding according to anaemia severity.**

| Anaemia severity | Number of events | Incidence rate per 100 patient-years (95% CI) | P-value | Multivariable HR (95% CI) |
| --- | --- | --- | --- | --- |
| Composite outcome (overall cohort) |  |  |  |  |
| No anaemia | 565 | 4.64 (4.27–5.04) | — | Reference |
| Moderate anaemia | 455 | 9.99 (9.10–10.95) | < 0.001 | 2.15 (1.90–2.44) |
| Severe anaemia | 86 | 16.05 (12.84–19.82) | < 0.001 | 3.46 (2.72–4.34) |
| Composite outcome (anaemic cohort) |  |  |  |  |
| Moderate anaemia | 455 | 9.99 (9.10–10.95) | — | Reference |
| Severe anaemia | 86 | 16.05 (12.84–19.82) | < 0.001 | 1.61 (1.26–2.03) |
| Major bleeding (overall cohort) |  |  |  |  |
| No anaemia | 110 | 0.88 (0.73–1.07) | — | Reference |
| Moderate anaemia | 81 | 1.75 (1.39–2.17) | < 0.001 | 1.97 (1.46–2.65) |
| Severe anaemia | 27 | 5.19 (3.42–7.55) | < 0.001 | 5.87 (3.70–9.01) |
| Major bleeding (anaemic cohort) |  |  |  |  |
| Moderate anaemia | 81 | 1.75 (1.39–2.17) | — | Reference |
| Severe anaemia | 27 | 5.19 (3.42–7.55) | < 0.001 | 2.97 (1.85–4.65) |

*Adjusted for age, female sex, body mass index, paroxysmal AF, heart failure, hypertension, diabetes, prior thromboembolic events, peripheral artery disease, cancer, dementia, coronary artery disease, oral anticoagulant use, and region of enrolment (APHRS vs EORP).*

**Supplementary Table 6. Baseline Characteristics of patients with and without Anaemia before and after Propensity Score Matching.**

| Variable | Pre-matching No anaemia  (n = 6463) | Pre-matching Anaemia  (n = 2741) | SMD | Post-matching No anaemia  (n = 2603) | Post-matching Anaemia  (n = 2603) | SMD |
| --- | --- | --- | --- | --- | --- | --- |
| Age (years, mean ± SD) | 66.4 ± 11.6 | 72.6 ± 10.4 | 0.565 | 72.1 ± 9.2 | 72.2 ± 10.4 | 0.004 |
| BMI (kg/m², mean ± SD) | 27.6 ± 5.0 | 26.9 ± 5.5 | 0.145 | 27.1 ± 4.8 | 27.0 ± 5.5 | 0.005 |
| Female sex (%) | 4488 (69.4) | 1196 (43.6) | 0.539 | 1188 (45.6) | 1177 (45.2) | 0.008 |
| European cohort (%) | 4646 (71.9) | 1932 (70.5) | 0.031 | 1869 (71.8) | 1869 (71.8) | <0.001 |
| OAC use (%) | 5657 (87.5) | 2298 (83.8) | 0.106 | 2247 (86.3) | 2204 (84.7) | 0.047 |
| Paroxysmal AF (%) | 2070 (32.0) | 877 (32.0) | 0.001 | 811 (31.2) | 832 (32.0) | 0.017 |
| Hypertension (%) | 3823 (59.2) | 1781 (65.0) | 0.120 | 1662 (63.8) | 1682 (64.6) | 0.016 |
| Diabetes (%) | 1296 (20.1) | 841 (30.7) | 0.246 | 765 (29.4) | 762 (29.3) | 0.003 |
| Heart failure (%) | 1851 (28.6) | 1212 (44.2) | 0.328 | 1088 (41.8) | 1126 (43.3) | 0.030 |
| Coronary artery disease (%) | 1415 (21.9) | 874 (31.9) | 0.227 | 800 (30.7) | 808 (31.0) | 0.007 |
| Peripheral artery disease (%) | 343 (5.3) | 238 (8.7) | 0.133 | 216 (8.3) | 225 (8.6) | 0.012 |
| Cancer (%) | 76 (1.2) | 113 (4.1) | 0.184 | 67 (2.6) | 88 (3.4) | 0.047 |
| Dementia (%) | 52 (0.8) | 64 (2.3) | 0.123 | 40 (1.5) | 51 (2.0) | 0.032 |
| Chronic kidney disease (%) | 489 (7.6) | 501 (18.3) | 0.323 | 359 (13.8) | 438 (16.8) | 0.084 |
| Thromboembolic events (%) | 712 (11.0) | 363 (13.2) | 0.068 | 348 (13.4) | 341 (13.1) | 0.008 |

*Abbreviations: AF, atrial fibrillation; BMI, body mass index; CKD, chronic kidney disease; OAC, oral anticoagulant; PSM, propensity score matching.*

**Supplementary Table 7. Interaction between Haemoglobin levels and Ethnicity for clinical outcomes (Multivariable RCS Models)**

| Outcome | Haemoglobin (overall effect) | Non-linearity | Ethnicity (overall effect) | Hb × Ethnicity interaction | Overall model |
| --- | --- | --- | --- | --- | --- |
| ****Composite outcome**** | <0.0001 | 0.029 | <0.0001 | 0.149 | <0.0001 |
| ****Major bleeding**** | <0.0001 | 0.333 | 0.1303 | 0.099 | <0.0001 |
| ****MACE**** | 0.0001 | 0.212 | <0.0001 | 0.313 | <0.0001 |

**Supplementary Table 8. Baseline characteristics of anaemic patients according to enrolment setting.**

| Characteristic | European N = 2,385 | Asian N = 987 | p-value |
| --- | --- | --- | --- |
| Age | 73 ± 10 | 73 ± 11 | 0.080 |
| Female | 1,393 (58.4%) | 557 (56.4%) | 0.300 |
| BMI | 28.0 ± 5.4 | 24.3 ± 4.6 | <0.001 |
| Systolic | 131 ± 21 | 130 ± 20 | 0.090 |
| Diastolic | 77 ± 12 | 71 ± 12 | <0.001 |
| Heart Rate | 83 ± 24 | 76 ± 16 | <0.001 |
| Hypertension | 1,537 (64.4%) | 668 (67.7%) | 0.120 |
| Diabetes mellitus | 723 (30.3%) | 307 (31.1%) | 0.700 |
| Lipid disorder | 928 (38.9%) | 428 (43.4%) | 0.100 |
| Smoking | 123 (5.2%) | 44 (4.5%) | 0.500 |
| Dementia | 56 (2.3%) | 36 (3.6%) | 0.047 |
| Heart failure | 1,239 (52.0%) | 298 (30.2%) | <0.001 |
| Valvular disease | 1,448 (60.7%) | 571 (57.9%) | 0.100 |
| Coronary artery disease | 781 (32.7%) | 242 (24.5%) | <0.001 |
| Peripheral vascular disease | 266 (11.2%) | 15 (1.5%) | <0.001 |
| Pulmonary arterial hypertension | 248 (10.4%) | 69 (7.0%) | <0.001 |
| Aortic plaque | 110 (4.6%) | 23 (2.3%) | <0.001 |
| COPD | 299 (12.5%) | 27 (2.7%) | <0.001 |
| CKD | 501 (21.0%) | 132 (13.4%) | <0.001 |
| Ischaemic Stroke | 178 (7.5%) | 84 (8.5%) | 0.006 |
| Thromboembolic events | 322 (13.5%) | 138 (14.0%) | 0.800 |
| Pulmonary Embolism/DVT | 63 (2.6%) | 9 (0.9%) | <0.001 |
| Clinically relevant non-major bleeding | 95 (4.0%) | 48 (4.9%) | <0.001 |
| Haemorrhagic events | 42 (1.8%) | 3 (0.3%) | 0.001 |
| Aspirin or other antiplatelet drugs | 640 (26.8%) | 278 (28.2%) | 0.700 |
| CHA₂DS₂-VASc | 3.89 ± 1.67 | 3.58 ± 1.62 | <0.001 |
| HAS-BLED | 625 (26.2%) | 218 (22.1%) | 0.014 |
| Rhy | 672 (28.2%) | 269 (27.3%) | 0.019 |
| Cancer | 90 (3.8%) | 45 (4.6%) | 0.400 |
| Paroxysmal AF | 610 (25.6%) | 418 (42.4%) | <0.001 |
| VKAs | 1,331 (55.8%) | 243 (24.6%) | <0.001 |
| OACs | 1,982 (83.1%) | 832 (84.3%) | 0.400 |
| NOACs | 653 (27.4%) | 589 (59.7%) | <0.001 |
| Dabigatran | 126 (19.3%) | 116 (19.7%) |  |
| Rivaroxaban | 305 (46.7%) | 173 (29.4%) |  |
| Apixaban | 214 (32.8%) | 208 (35.3%) |  |
| Edoxaban | 8 (1.2%) | 92 (15.6%) |  |

*Abbreviations: OAC, oral anticoagulant; NOACs, non–vitamin K antagonist oral anticoagulant; VKAs, vitamin K antagonist; BMI, body mass index; CKD, chronic kidney disease; COPD, chronic obstructive pulmonary disease; AF, atrial fibrillation*

**Supplementary Table 9. Univariable and multivariable logistic regression for factors associated with enrolment setting.**

| **Variable** | **Univariable OR (95% CI)** | **p** | **Multivariable OR (95% CI)** | **p** |
| --- | --- | --- | --- | --- |
| **Age** | 1.007 (0.999–1.014) | 0.073 | 0.987 (0.977–0.997) | 0.015 |
| **Female** | 0.92 (0.79–1.07) | 0.291 | 0.73 (0.60–0.91) | 0.004 |
| **BMI** | 0.85 (0.83–0.87) | <0.001 | 0.84 (0.82–0.86) | <0.001 |
| **Hypertension** | 1.14 (0.97–1.33) | 0.114 | 1.38 (1.10–1.72) | 0.0055 |
| **Diabetes** | 1.04 (0.89–1.22) | 0.627 | 1.56 (1.23–1.97) | <0.001 |
| **Dyslipidaemia** | 1.14 (0.98–1.33) | 0.089 | 1.39 (1.12–1.72) | 0.0028 |
| **Smoking** | 0.88 (0.62–1.25) | 0.482 | 0.94 (0.57–1.55) | 0.796 |
| **Dementia** | 1.57 (1.03–2.41) | 0.037 | 1.71 (0.89–3.29) | 0.105 |
| **Heart failure** | 0.41 (0.35–0.47) | <0.001 | 0.51 (0.41–0.64) | <0.001 |
| **CAD** | 0.61 (0.52–0.72) | <0.001 | 0.75 (0.59–0.96) | 0.020 |
| **PAD** | 0.12 (0.07–0.20) | <0.001 | 0.15 (0.08–0.29) | <0.001 |
| **COPD** | 0.20 (0.13–0.29) | <0.001 | 0.27 (0.16–0.45) | <0.001 |
| **CKD** | 0.58 (0.47–0.72) | <0.001 | 0.78 (0.58–1.05) | 0.103 |
| **Thromboembolic events** | 1.04 (0.84–1.29) | 0.728 | 1.09 (0.81–1.46) | 0.578 |
| **Cancer** | 1.21 (0.84–1.75) | 0.305 | 1.10 (0.65–1.87) | 0.710 |
| **Paroxysmal AF** | 2.09 (1.79–2.45) | <0.001 | 1.59 (1.28–1.97) | <0.001 |
| **Major bleeding** | 0.17 (0.05–0.55) | 0.003 | 0.29 (0.07–1.28) | 0.101 |
| **OACs** | 1.09 (0.89–1.33) | 0.410 | 1.19 (0.89–1.59) | 0.237 |
| **Rhythm control** | 0.81 (0.69–0.96) | 0.017 | 0.66 (0.53–0.83) | <0.001 |

*Abbreviations: AF, atrial fibrillation; BMI, body mass index; CAD, coronary artery disease; PAD, peripheral artery disease; CKD, chronic kidney disease; COPD, chronic obstructive pulmonary disease; OACs, oral anticoagulants; OR, odds ratio; CI, confidence interval*

**Supplementary Table 10.** **Incidence rates for the outcomes to enrolment setting.**

| Outcome | Events (European) | IR per 100 PY (95% CI) | Events (Asian) | IR per 100 PY (95% CI) | P-value |
| --- | --- | --- | --- | --- | --- |
| Composite outcome | 521 | 12.66 (11.59–13.79) | 20 | 2.06 (1.26–3.18) | <0.0001 |
| All-cause death | 404 | 9.43 (8.53–10.39) | 0 | 0 (0–0.38) | - |
| Cardiovascular death | 159 | 3.71 (3.16–4.33) | 0 | 0 (0–0.38) | - |
| MACE | 294 | 7.05 (6.27–7.91) | 20 | 2.06 (1.26–3.18) | <0.0001 |
| Acute coronary syndrome | 88 | 2.09 (1.68–2.58) | 8 | 0.82 (0.35–1.61) | 0.0059 |
| Thromboembolic events | 60 | 1.43 (1.09–1.84) | 12 | 1.23 (0.63–2.15) | 0.763 |
| Major bleeding | 87 | 2.08 (1.66–2.56) | 21 | 2.16 (1.34–3.30) | 0.902 |

*Statistical comparison was not performed for outcomes with no observed events in one group.*

**Supplementary Table 11.** **Adjusted hazard ratios for the outcomes according to enrolment setting.**

| Outcome | Univariable HR (95% CI) | p | Multivariable HR (95% CI) | p |
| --- | --- | --- | --- | --- |
| Composite outcome | 0.14 (0.09–0.23) | <0.0001 | 0.13 (0.07–0.22) | <0.0001 |
| All-cause death | - | - | - | - |
| Cardiovascular death | - | - | - | - |
| MACE | 0.25 (0.16–0.40) | <0.0001 | 0.24 (0.13–0.42) | <0.0001 |
| Acute coronary syndrome | 0.36 (0.17–0.77) | 0.008 | 0.23 (0.08–0.66) | 0.006 |
| Thromboembolic events | 0.81 (0.42–1.56) | 0.534 | 0.81 (0.37–1.79) | 0.599 |
| Major bleeding | 0.74 (0.45–1.21) | 0.23 | 0.65 (0.36–1.17) | 0.150 |

Statistical comparison was not performed for outcomes with no observed events in one group.

Adjusted for age, female sex, body mass index, paroxysmal AF, heart failure, hypertension, diabetes, prior thromboembolic events, peripheral artery disease, cancer, dementia, coronary artery disease, oral anticoagulant use, and region of enrolment (APHRS vs EORP).

**Supplementary Table 12. Baseline characteristics of anaemic patients according to anticoagulation status and type of oral anticoagulant (NOACs vs VKAs).**

| Characteristic | Anaemia + OACs (N = 2,814) | Anaemia + No OACs (N = 557) | P value | Anaemia + NOACs (N = 1,238) | Anaemia + VKAs (N = 1,571) | P value |
| --- | --- | --- | --- | --- | --- | --- |
| Age (years) | 73 ± 10 | 72 ± 13 | 0.038 | 74 ± 10 | 73 ± 10 | 0.005 |
| Female | 1,635 (58.1%) | 314 (56.4%) | 0.453 | 731 (59.0%) | 900 (57.3%) | 0.356 |
| European | 1,982 (70.4%) | 402 (72.2%) | 0.445 | 649 (52.4%) | 1,328 (84.6%) | <0.001 |
| Asian | 832 (29.6%) | 155 (27.8%) |  | 589 (47.6%) | 243 (15.5%) |  |
| BMI (kg/m²) | 27.0 ± 5.5 | 26.8 ± 5.4 | 0.574 | 26.5 ± 5.6 | 27.4 ± 5.4 | <0.001 |
| SBP (mmHg) | 130 ± 20 | 132 ± 22 | 0.182 | 130 ± 21 | 130 ± 20 | 0.924 |
| DBP (mmHg) | 75 ± 12 | 75 ± 13 | 0.712 | 74 ± 13 | 75 ± 12 | 0.012 |
| HR (bpm) | 81 ± 22 | 82 ± 24 | 0.813 | 79 ± 21 | 83 ± 23 | <0.001 |
| Hypertension | 1,857 (66.0%) | 348 (62.5%) | 0.140 | 796 (64.3%) | 1,058 (67.4%) | 0.090 |
| Diabetes | 860 (30.6%) | 170 (30.5%) | 0.920 | 371 (30.0%) | 486 (30.9%) | 0.563 |
| Dyslipidaemia | 1,142 (40.6%) | 213 (38.2%) | 0.415 | 484 (39.1%) | 658 (41.9%) | 0.109 |
| Smoking | 129 (4.6%) | 38 (6.8%) | 0.031 | 47 (3.8%) | 82 (5.2%) | 0.084 |
| Dementia | 59 (2.1%) | 33 (5.9%) | <0.001 | 26 (2.1%) | 33 (2.1%) | >0.999 |
| Heart failure | 1,295 (46.0%) | 242 (43.4%) | 0.282 | 445 (36.0%) | 847 (53.9%) | <0.001 |
| Valvular disease | 1,727 (61.4%) | 292 (52.4%) | <0.001 | 650 (52.5%) | 1,075 (68.4%) | <0.001 |
| CAD | 827 (29.4%) | 196 (35.2%) | 0.009 | 288 (23.3%) | 538 (34.3%) | <0.001 |
| PAD | 240 (8.5%) | 41 (7.4%) | 0.401 | 64 (5.2%) | 174 (11.1%) | <0.001 |
| Pulmonary hypertension | 266 (9.5%) | 51 (9.2%) | 0.535 | 79 (6.4%) | 185 (11.8%) | <0.001 |
| Aortic plaque | 116 (4.1%) | 17 (3.1%) | 0.398 | 42 (3.4%) | 74 (4.7%) | 0.022 |
| COPD | 278 (9.9%) | 48 (8.6%) | 0.389 | 83 (6.7%) | 194 (12.3%) | <0.001 |
| CKD | 506 (18.0%) | 127 (22.8%) | 0.009 | 168 (13.6%) | 337 (21.5%) | <0.001 |
| Prior Ischaemic stroke | 228 (8.1%) | 34 (6.1%) | 0.234 | 98 (7.9%) | 130 (8.3%) | 0.378 |
| Prior Thromboembolic events | 409 (14.5%) | 51 (9.2%) | <0.001 | 172 (13.9%) | 236 (15.0%) | 0.418 |
| Cancer | 102 (3.6%) | 33 (5.9%) | 0.017 | 55 (4.4%) | 46 (2.9%) | 0.041 |
| Paroxysmal AF | 793 (28.2%) | 234 (42.0%) | <0.001 | 454 (36.7%) | 336 (21.4%) | <0.001 |
| Intracranial haemorrhage | 32 (1.1%) | 22 (4.0%) | <0.001 | 19 (1.5%) | 13 (0.8%) | 0.448 |
| Major extracranial bleeding | 118 (4.2%) | 42 (7.5%) | 0.002 | 63 (5.1%) | 54 (3.4%) | 0.003 |
| Clinically relevant non-major bleeding | 113 (4.0%) | 30 (5.4%) | 0.240 | 52 (4.2%) | 61 (3.9%) | <0.001 |
| Antiplatelet drugs | 654 (23.2%) | 264 (47.4%) | <0.001 | 291 (23.5%) | 362 (23.0%) | 0.958 |
| CHA₂DS₂-VASc | 3.85 ± 1.63 | 3.56 ± 1.83 | 0.003 | 3.68 ± 1.58 | 3.97 ± 1.65 | <0.001 |
| HAS-BLED ≥ 3 | 661 (23.5%) | 182 (32.7%) | <0.001 | 187 (15.1%) | 472 (30.1%) | <0.001 |
| Rhythm Control | 792 (28.1%) | 148 (26.6%) | 0.505 | 402 (32.5%) | 389 (24.8%) | <0.001 |

*Abbreviations: OAC, oral anticoagulant; NOACs, non–vitamin K antagonist oral anticoagulant; VKAs, vitamin K antagonist; BMI, body mass index; CKD, chronic kidney disease; COPD, chronic obstructive pulmonary disease; AF, atrial fibrillation*

**Supplementary Table 13. Incidence rates and adjusted hazard ratios for the composite outcome and major bleeding according to exploratory analyses.**

| Anaemia + OAC status | Number of events | Incidence rate per 100 patient-years (95% CI) | P-value | Multivariable HR (95% CI) |
| --- | --- | --- | --- | --- |
| OAC use | | | | |
| Composite outcome | | | | |
| Anaemia + OAC | 420 | 9.76 (8.85 – 10.74) | < 0.0001 | 0.61 (0.48-0.78) |
| Anaemia + No OAC | 121 | 15.41 (12.79 – 18.42) | Reference | Reference |
| Major bleeding | | | | |
| Anaemia + OACs | 89 | 2.05 (1.65 – 2.52) | 0.599 | 0.80 (0.47 – 1.39) |
| Anaemia + No OACs | 19 | 2.32 (1.40 – 3.63) | Reference | Reference |
| Anaemia status (VKAs versus NOACs) | | | | |
| Composite outcome | | | | |
| Anaemia + NOACs | 129 | 7.38 (6.16–8.77) | Reference | Reference |
| Anaemia + VKAs | 291 | 11.42 (10.15–12.82) | < 0.0001 | 1.06 ( 0.83- 1.35) |
| Major bleeding | | | | |
| Anaemia + NOACs | 32 | 1.82 (1.25–2.57) | Reference | Reference |
| Anaemia + VKAs | 57 | 2.21 (1.68–2.87) | 0.391 | 1.20 (0.72-2.01) |

Adjusted for age, female sex, body mass index, paroxysmal AF, heart failure, hypertension, diabetes, prior thromboembolic events, peripheral artery disease, cancer, dementia, coronary artery disease, oral anticoagulant use, and region of enrolment (APHRS vs EORP).

**Supplementary Table 14. Global and interaction tests for haemoglobin and anticoagulant type in anaemic patients**

| Effect | χ² | P value |
| --- | --- | --- |
| Haemoglobin (overall effect) | 15.22 | 0.019 |
| Anticoagulant type (VKAs vs NOACs) | 1.59 | 0.811 |
| Haemoglobin × anticoagulant type (interaction) | 1.54 | 0.673 |
| Non-linear interaction | 1.54 | 0.463 |

**Supplementary Table 15. VKAs versus NOACs according to haemoglobin levels in anaemic patients**

| Hb (g/dL) | HR (VKAs vs NOACs) | 95% CI |
| --- | --- | --- |
| 8 | 1.83 | 0.99 – 3.38 |
| 10 | 1.49 | 1.05 – 2.12 |
| 12 | 0.98 | 0.95 – 1.01 |

**Supplementary Table 16. Fine–Gray competing risk analysis for non-fatal cardiovascular events**

| Variable | sHR | 95% CI | P value |
| --- | --- | --- | --- |
| Anaemia | 1.01 | 0.79–1.29 | 0.93 |
| Age (per year) | 1.01 | 1.00–1.02 | 0.014 |
| BMI (per kg/m²) | 0.99 | 0.96–1.01 | 0.23 |
| Female sex | 1.06 | 0.85–1.33 | 0.61 |
| Paroxysmal AF | 1.20 | 0.95–1.52 | 0.12 |
| Heart failure | 1.14 | 0.90–1.43 | 0.27 |
| Hypertension | 1.02 | 0.81–1.29 | 0.84 |
| Diabetes mellitus | 1.20 | 0.94–1.53 | 0.14 |
| Prior thromboembolic events | **1.56** | **1.17–2.08** | **0.002** |
| Peripheral artery disease | 0.78 | 0.52–1.15 | 0.21 |
| Cancer | 1.03 | 0.52–2.03 | 0.94 |
| Dementia | 0.57 | 0.21–1.57 | 0.28 |
| Coronary artery disease | **2.44** | **1.93–3.08** | **<0.001** |
| Oral anticoagulant use | **0.59** | **0.45–0.77** | **<0.001** |
| Ethnicity (APHRS vs EORP) | **0.49** | **0.34–0.70** | **<0.001** |

**Supplementary Figure 1. Flow chart**


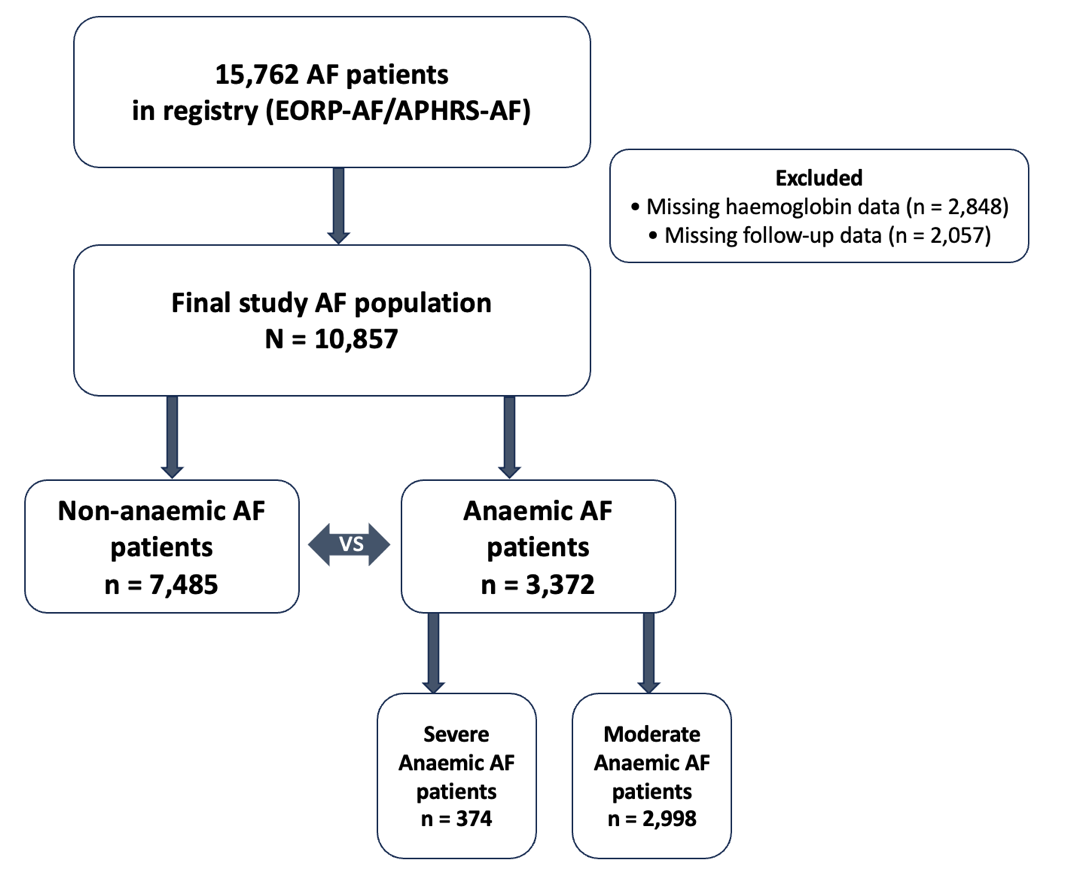


**Supplementary Figure 2. Kaplan–Meier Curves for the Secondary Outcomes According to Anaemia.**

***Legend:*** *Kaplan–Meier survival curves comparing patients with and without anaemia for secondary outcomes. Survival probability is shown over a 2-year follow-up; p-values from log-rank tests indicate between-group differences.*

**Supplementary Figure 3. Sensitivity Analysis: Kaplan–Meier curves and multivariable cox model for the Major bleeding according to anaemia severity**


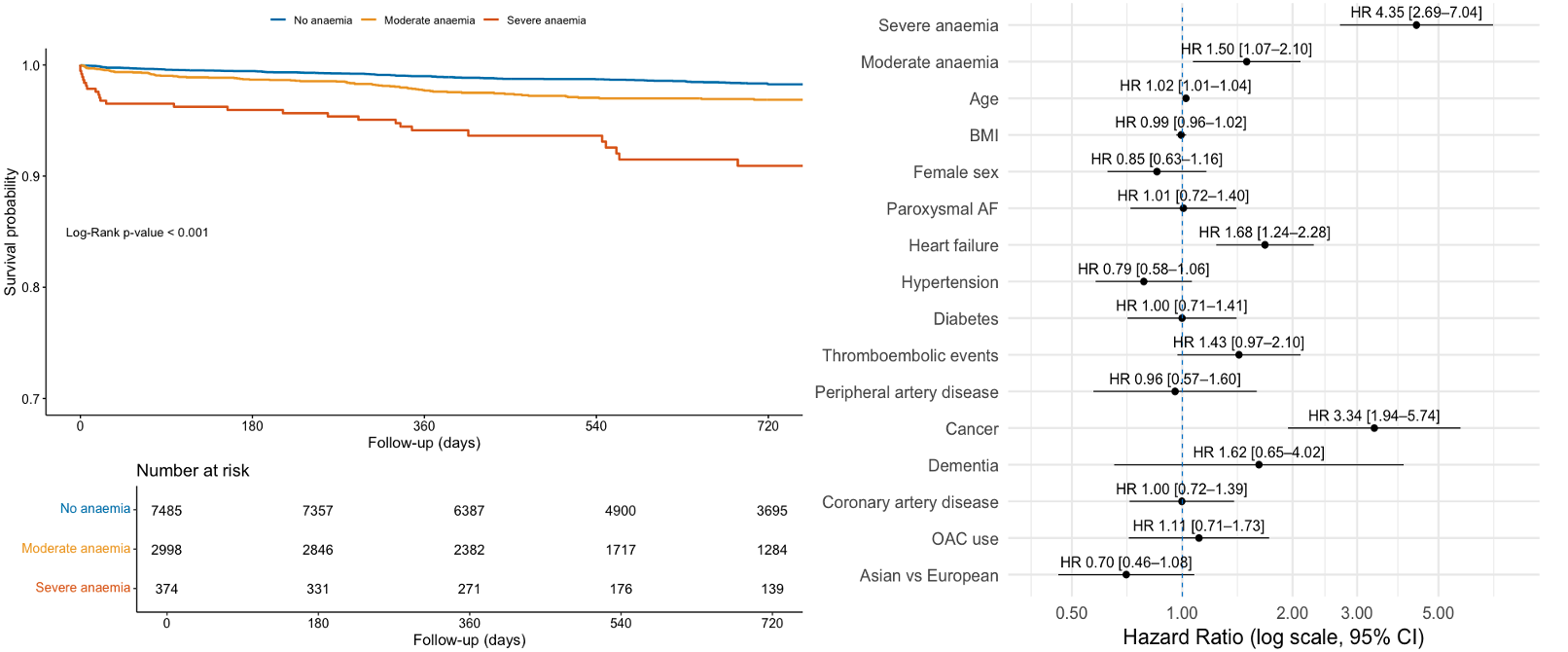


*The left panel shows Kaplan–Meier estimates of major bleeding–free survival by anaemia severity (no anaemia, blue; moderate anaemia, orange; severe anaemia, red). Differences between groups were assessed using the log-rank test. The right panel displays adjusted hazard ratios (HRs) with 95% confidence intervals (CIs) for major bleeding derived from the multivariable Cox regression model including anaemia severity and all listed covariates. Abbreviations: AF, atrial fibrillation; BMI, body mass index; CI, confidence interval; HR, hazard ratio; OAC, oral anticoagulant.*

**Supplementary Figure 4. Covariate Balance Before and After Propensity Score Matching (PSM)**

**
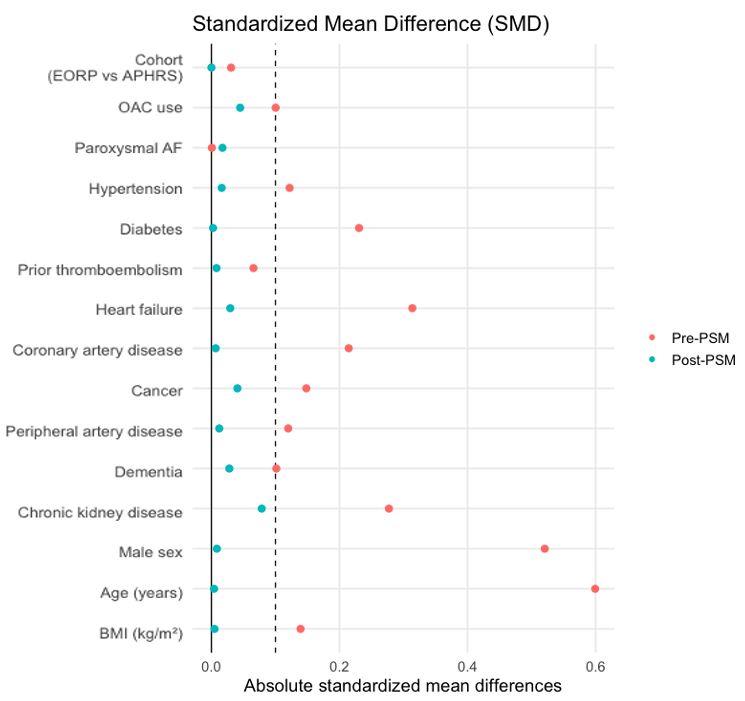
**

*Standardized mean differences (SMDs) for baseline covariates before (red) and after (blue) propensity score matching. The dashed vertical line at 0.1 indicates the threshold for acceptable covariate balance.* *After matching, all variables achieved adequate balance (SMD < 0.1).*

**Supplementary Figure 5. Exploratory Analysis: Kaplan–Meier Curves and Multivariable Cox Model for the Composite Outcome according to anticoagulation status (OACs vs no-OACs).**


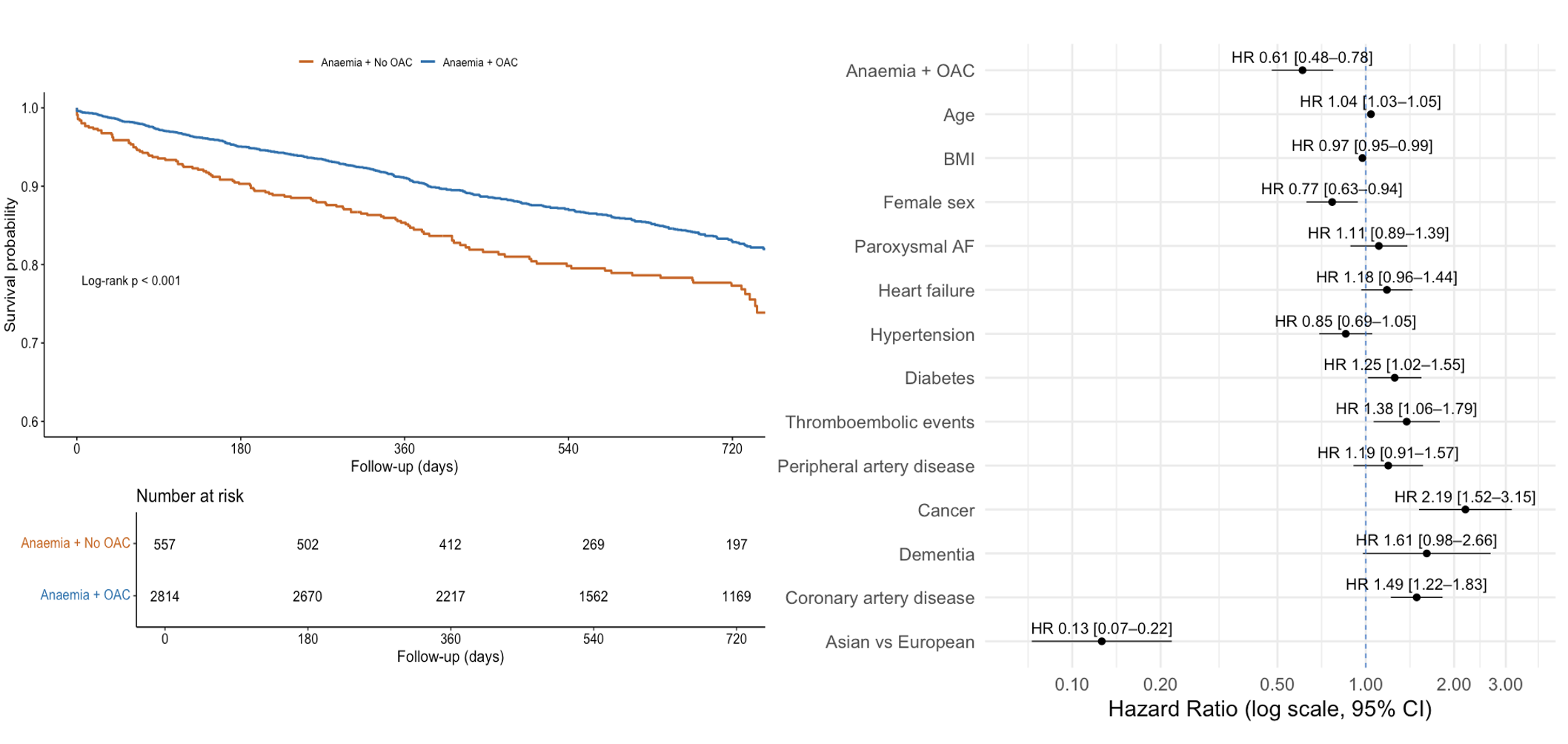


*Kaplan–Meier curve (left) showing survival from the composite outcome according to oral anticoagulant (OAC) use among patients with anaemia. The orange line represents patients not receiving OACs, and the blue line those treated with OACs (log-rank p < 0.001). Forest plot (right) presents the multivariable Cox regression for the composite outcome in anaemic patients, adjusted for major clinical covariates. Hazard ratios (HR) and 95% confidence intervals (CI) are displayed on a logarithmic scale.*

**Supplementary Figure 6. Exploratory Analysis: Kaplan–Meier Curves and Multivariable Cox Model for the Major bleeding according to anticoagulation status (OACs vs no-OACs).**

**
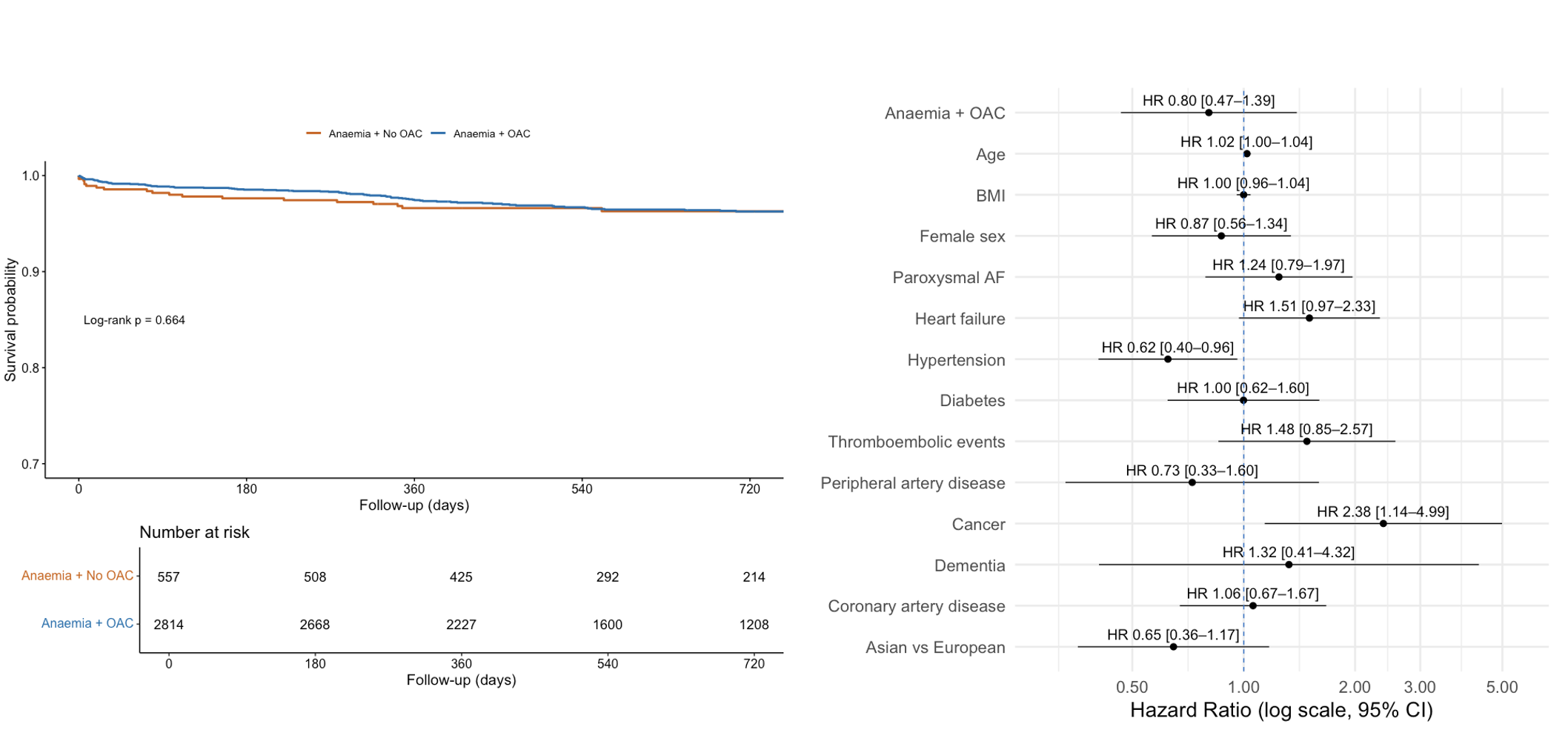
**

*Kaplan–Meier curve (left) showing survival free from the Major Bleeding according to oral anticoagulant (OAC) use among patients with anaemia. The orange line represents patients not receiving OACs, and the blue line those treated with OACs (log-rank p = 0.664). Forest plot (right) presents the multivariable Cox regression for the composite outcome in anaemic patients, adjusted for major clinical covariates. Hazard ratios (HR) and 95% confidence intervals (CI) are displayed on a logarithmic scale.*

**Supplementary Figure 7. Exploratory Analysis: Kaplan–Meier Curves and Multivariable Cox Model for the Composite outcome according to type of anticoagulant (NOACs vs VKAs).**


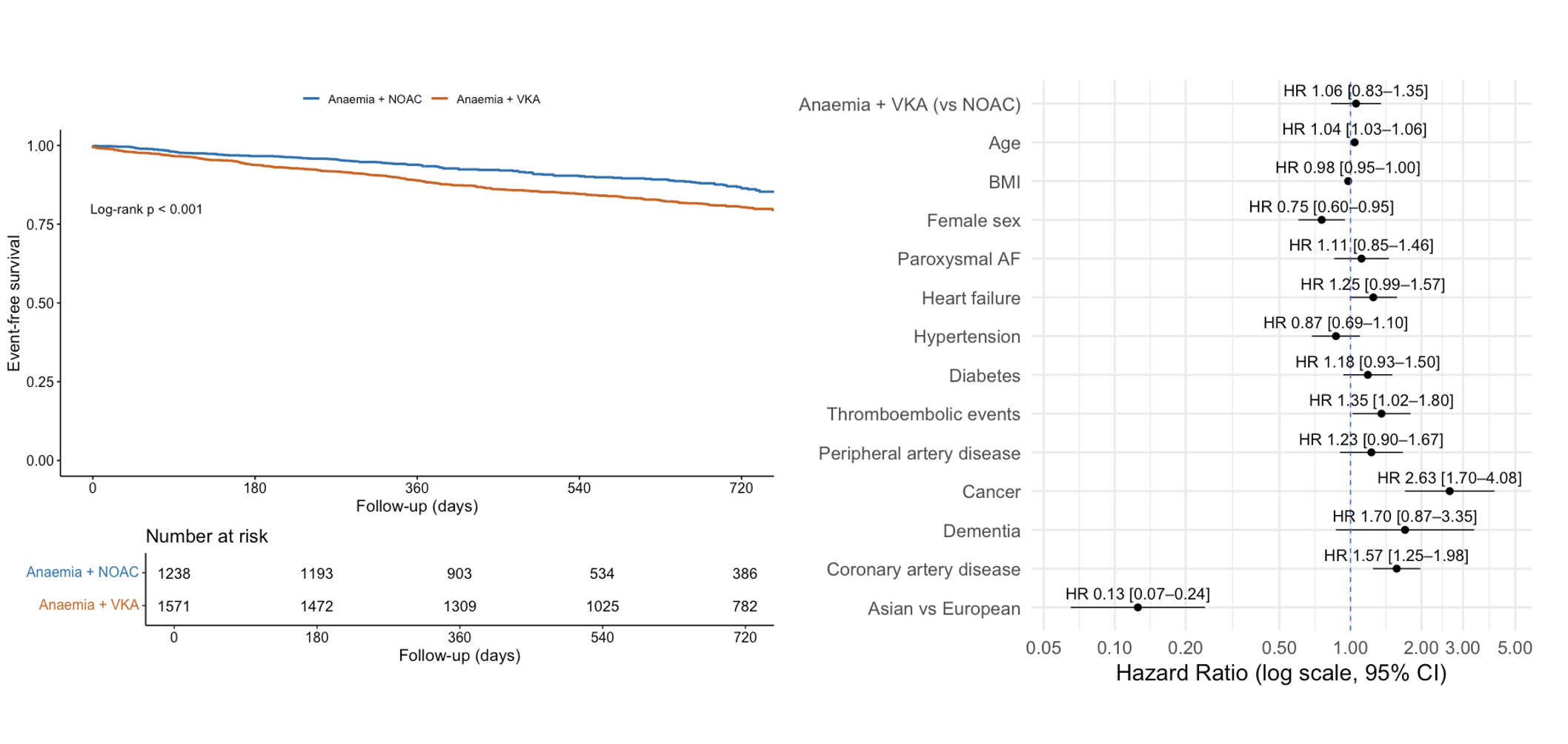


*Kaplan–Meier curve (left) showing survival free from the composite outcome according to anticoagulant type among patients with anaemia. The orange line represents patients treated with VKAs, and the blue line those receiving NOACs (log-rank p < 0.001). Forest plot (right) presents the multivariable Cox regression for the composite outcome in anaemic patients, adjusted for major clinical covariates. Hazard ratios (HR) and 95% confidence intervals (CI) are displayed on a logarithmic scale.*

**Supplementary Figure 8. Exploratory Analysis: Kaplan–Meier Curves and Multivariable Cox Model for the major bleeding according to type of anticoagulant (NOACs vs VKAs).**


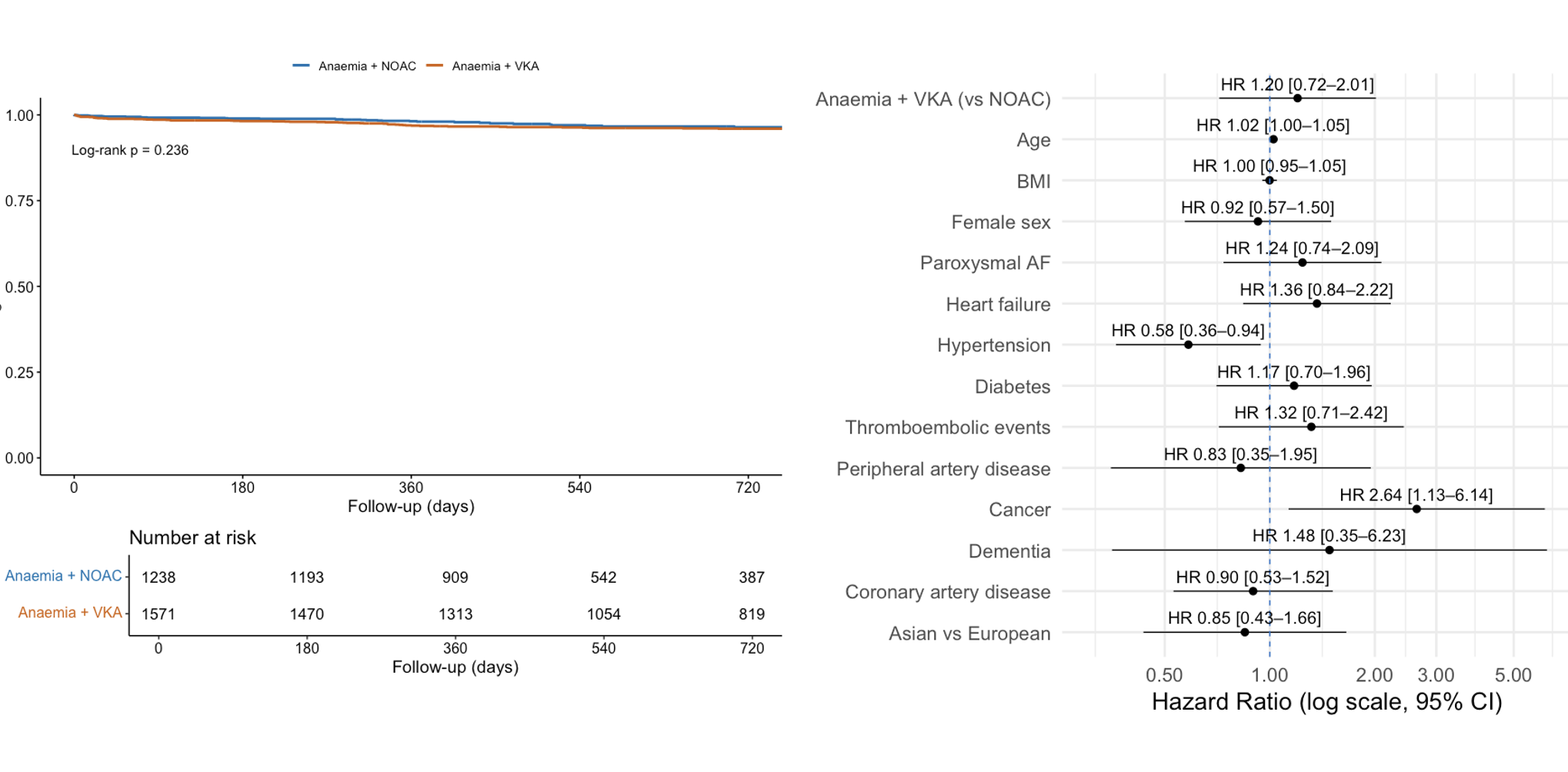


*Kaplan–Meier curve (left) showing the cumulative incidence of major bleeding according to anticoagulant type among patients with anaemia. The orange line represents patients treated with VKAs, and the blue line those receiving NOACs (log-rank p = 0.236).Forest plot (right) presents the multivariable Cox regression for major bleeding in anaemic patients, adjusted for major clinical covariates. Hazard ratios (HR) and 95% confidence intervals (CI) are displayed on a logarithmic scale.*

**Supplementary Figure 9. Association between haemoglobin levels and composite outcome according to anticoagulant type in anaemic patients (NOACs vs VKAs).**


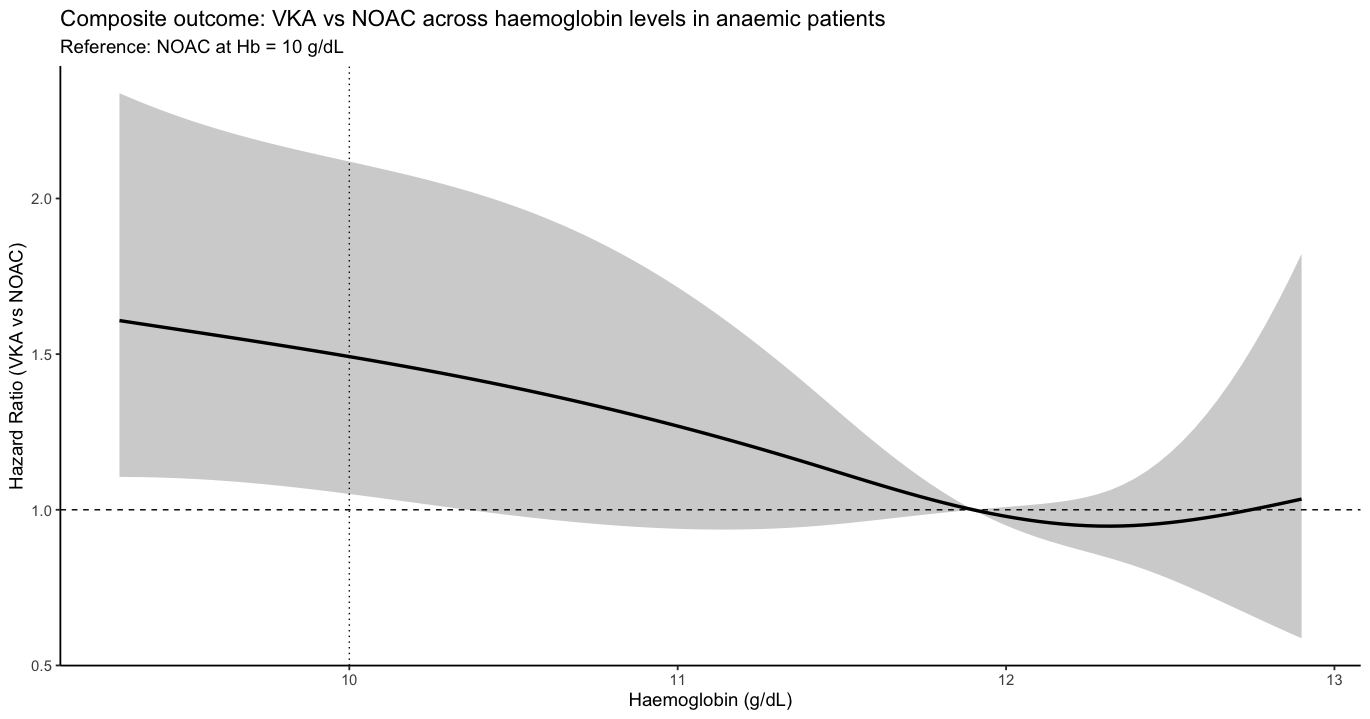


*Restricted cubic spline models showing adjusted hazard ratios (HRs) with 95% confidence intervals (CIs) for the association between haemoglobin levels and the composite outcome in anaemic patients, according to anticoagulant type*

**Supplementary Figure 10.** **Competing risk analysis of fatal and non-fatal cardiovascular events**

**
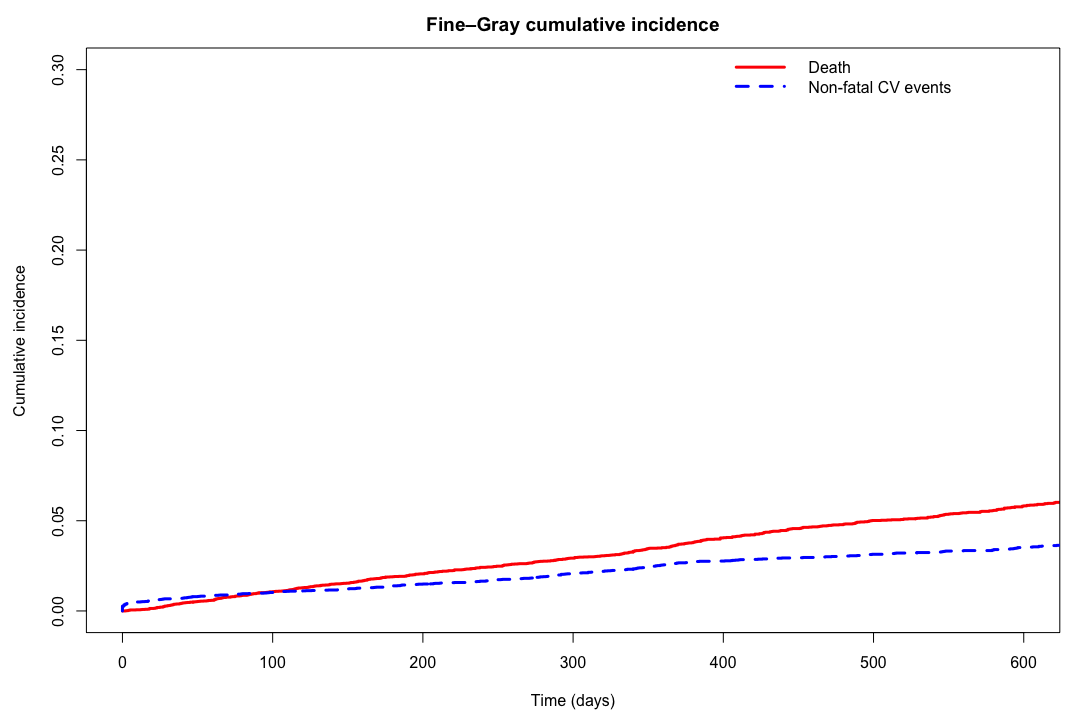
**

*Cumulative incidence curves for all-cause death (red solid line) and non-fatal cardiovascular events (blue dashed line) estimated using the Fine–Gray competing risk model. Non-fatal cardiovascular events were defined as the first occurrence of acute coronary syndrome or thromboembolic events. Death was treated as the competing event.*

**Supplementary Figure 11. Effect of Anaemia on the Composite Outcome in Prespecified Subgroups.**

**
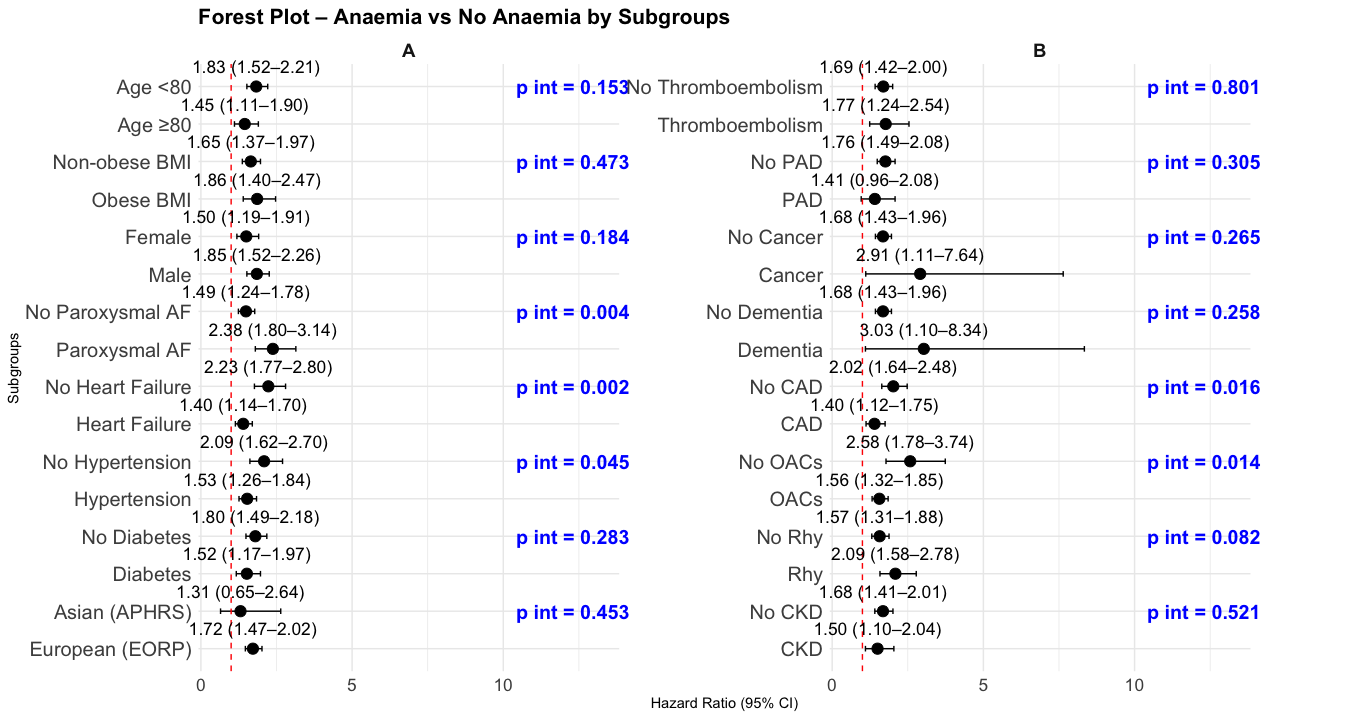
**

*Forest plot showing the association between anaemia and the composite outcome across clinical subgroups. Hazard ratios (95% CI) are adjusted for baseline covariates. p int indicates the p value for interaction between anaemia status and each subgroup variable.*
